# Targeting TMEM176B Enhances Antitumor Immunity and Augments the Efficacy of Immune Checkpoint Blockers by Unleashing Inflammasome Activation

**DOI:** 10.1016/j.ccell.2019.04.003

**Published:** 2019-05-13

**Authors:** Mercedes Segovia, Sofia Russo, Mathias Jeldres, Yamil D. Mahmoud, Valentina Perez, Maite Duhalde, Pierre Charnet, Matthieu Rousset, Sabina Victoria, Florencia Veigas, Cédric Louvet, Bernard Vanhove, R. Andrés Floto, Ignacio Anegon, Maria Cristina Cuturi, M. Romina Girotti, Gabriel A. Rabinovich, Marcelo Hill

**Affiliations:** 1 Laboratory of Immunoregulation and Inflammation, Institut Pasteur de Montevideo, 11400 Montevideo, Uruguay; 2 Immunobiology Department, Faculty of Medicine, University of the Republic, 11800 Montevideo, Uruguay; 3 Laboratories of Immunopathology and Translational Immuno-Oncology, Institute of Biology and Experimental Medicine (IBYME), National Council of Scientific and Technical Investigations (CONICET), C1428 Buenos Aires, Argentina; 4 Institut des Biomolécules Max Mousseron (IBMM), UMR 5247, CNRS ENSCM, Université de Montpellier, 34093 Montpellier, France; 5 INSERM UMR 1064, Center for Research in Transplantation and Immunology, Université de Nantes, CHU Nantes, Institut de Transplantation Urologie Néphrologie (ITUN), 44093 Nantes, France; 6 Xenothera, 44093 Nantes, France; 7 Molecular Immunity Unit, Department of Medicine, University of Cambridge, CB2 0QH Cambridge, UK; 8 Department of Biological Chemistry, School of Exact and Natural Sciences, University of Buenos Aires, C1428 Buenos Aires, Argentina

**Keywords:** TMEM176B, immune checkpoint blockers, cancer, inflammasome, dendritic cells, ion channel

## Abstract

Although immune checkpoint blockers have yielded significant clinical benefits in patients with different malignancies, the efficacy of these therapies is still limited. Here, we show that disruption of transmembrane protein 176B (TMEM176B) contributes to CD8^+^ T cell-mediated tumor growth inhibition by unleashing inflammasome activation. Lack of *Tmem176b* enhances the antitumor activity of anti-CTLA-4 antibodies through mechanisms involving caspase-1/IL-1β activation. Accordingly, patients responding to checkpoint blockade therapies display an activated inflammasome signature. Finally, we identify BayK8644 as a potent TMEM176B inhibitor that promotes CD8^+^ T cell-mediated tumor control and reinforces the antitumor activity of both anti-CTLA-4 and anti-PD-1 antibodies. Thus, pharmacologic de-repression of the inflammasome by targeting TMEM176B may enhance the therapeutic efficacy of immune checkpoint blockers.

## Highlights

• TMEM176B inhibits the NLRP3 inflammasome by controlling cytosolic Ca^2+^.
• Lack of *Tmem176b* enhances antitumor immunity via the caspase-1/IL-1β pathway
• Human tumors responding to immune checkpoint blockade display an inflammasome-activated signature
• A TMEM176B inhibitor improves the antitumor activity of immune checkpoint blockade

## Teaser

Segovia et al. show that TMEM176B inhibits inflammasome activation and IL-1β cleavage by controlling cytosolic Ca^2+^ in dendritic cells and macrophages. Inhibition of TMEM176B enhances tumor infiltration by CD8^+^ T cells and improves the antitumor activity of immune checkpoint blockers.

## Significance

**Therapies targeting immune checkpoint pathways have revolutionized treatment of several cancers. However, innate and adaptive mechanisms may limit the clinical efficacy of this therapeutic modality. Here, we identify the transmembrane protein 176B (TMEM176B) as an innate immune checkpoint that curtails CD8**^**+**^ **T cell-mediated immunity by repressing inflammasome activation. Genetic disruption or pharmacologic inhibition of TMEM176B potentiates antitumor immunity and enhances the efficacy of anti-CTLA-4 and anti-PD-1 antibodies in mice by unleashing inflammasome activation. Accordingly, an activated inflammasome signature delineates favorable clinical responses in patients receiving immune checkpoint blockers. Thus, targeting TMEM176B may influence antitumor effector mechanisms by de-repressing inflammasome activation.**

## Introduction

Blockade of immune checkpoints, including the cytotoxic T lymphocyte-associated protein (CTLA)-4 and the programmed cell death-1 (PD-1)/PD ligand-1 (PD-L1) pathway, have increased overall survival and progression-free survival of cancer patients. However, only a restricted number of patients show clinical benefits (Syn et al., 2017, Binnewies et al., 2018), suggesting that other immune inhibitory mechanisms may limit the efficacy of these treatments. In this regard, high intratumoral K^+^ leads to T cell dysfunction by inhibiting voltage and Ca^2+^-dependent K^+^ channels expressed in antitumoral T lymphocytes (Eil et al., 2016), suggesting a role for ionic channels as regulatory checkpoints and therapeutic targets to reinforce antitumor immunity.

Recognition of immunogenic tumors by innate immune sensors including the TMEM173 (STING) type I interferon pathway leads to stimulation of CD8^+^ T cell-mediated immunity and potentiation of CTLA-4- and PD-1-targeted therapies (Woo et al., 2014). Within the human myeloid compartment, STING controls the NLRP3 inflammasome (Gaidt et al., 2017), a cytosolic multiprotein complex that, once activated, cleaves caspase-1, which then processes pro-interleukin-1β (IL-1β) and pro-IL-18 to give the active and secreted forms of these pro-inflammatory cytokines (Rathinam and Fitzgerald, 2016). Altered levels of cytosolic cations have been shown to control secretion of active IL-1β through modulation of inflammasome activation (Gong et al., 2018). Interestingly, activation of the NLRP3 inflammasome following immunogenic chemotherapy sensitizes tumors to immune checkpoint blockers (Pfirschke et al., 2016). However, the role of the NLRP3 inflammasome in modulating checkpoint blockade therapies has not yet been explored.

Transmembrane protein 176B (TMEM176B), also known as tolerance-related and induced (TORID), has been identified as an immunoregulatory cation channel (Louvet et al., 2005, Segovia et al., 2014). This ubiquitously expressed protein contains four transmembrane domains and an ITIM motif in its C terminus (Eon Kuek et al., 2016). TMEM176B and its homologous TMEM176A are members of the CD20-like MS4A family of proteins (Eon Kuek et al., 2016, Louvet et al., 2005) and are highly expressed in monocytes, macrophages, and CD11b^+^ dendritic cells (DCs) (Condamine et al., 2010). Here we explored the role of TMEM176B in inflammasome regulation, T cell-dependent antitumor immunity and response to immune checkpoint blockade therapies.

## Results

### TMEM176B Inhibits Activation of the NLRP3 Inflammasome

**Figure 1:**
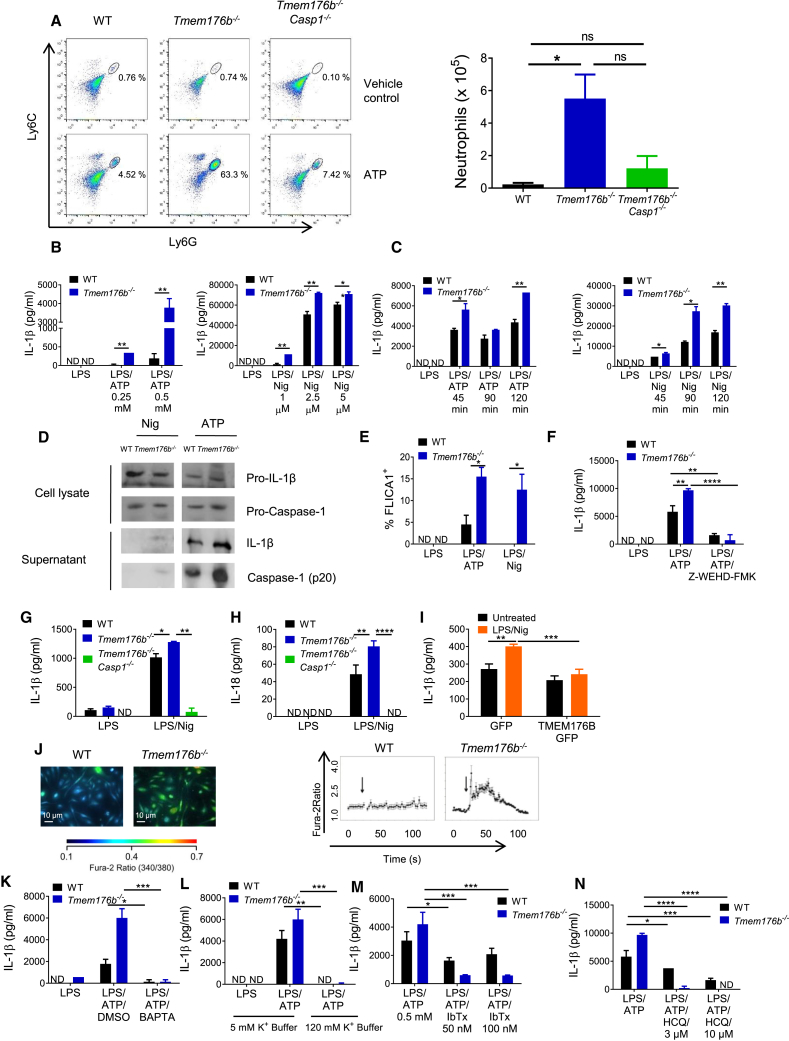
The Ionic Channel TMEM176B Inhibits the NLRP3 Inflammasome (A) Representative dot plots and absolute number of neutrophils (CD11b^+^ Ly6C^int^ Ly6G^+^) in peritoneal lavage 4 h after intraperitoneal (i.p.) injection with vehicle control (PBS) or 20 mg/kg ATP. In the plots, CD11b^+^ cells were analyzed for Ly6C and Ly6G expression. At least six animals were studied in each group in two independent experiments. ns, not significant; ^∗^p < 0.05; one-way ANOVA test. (B and C) Dose-response (B) and time-response (C) analysis of WT and *Tmem176b*^*−/−*^ bone marrow-derived DCs (BMDCs) treated with LPS (0.25 μg/mL) for 4 h, washed and treated with ATP (left) or nigericine (Nig) (right). IL-1β in culture supernatants was determined by ELISA. One experiment representative of five is shown. ^∗^p < 0.05, ^∗∗^p < 0.01; two-way ANOVA test. (D) Western blot analysis of pro-IL-1β and pro-caspase-1 (lysates) or IL-1β and caspase-1 (supernatants) in WT and *Tmem176b*^*−/−*^ BMDCs stimulated with LPS as in (B and C) and then treated for 90 min with 2.5 μM Nig or 0.5 mM ATP. One experiment representative of three is shown. (E) Caspase-1 activation in WT and *Tmem176b*^*−/−*^ BMDCs treated with LPS and then exposed to 0.5 mM ATP or 2.5 μM Nig for 45 min. Cells were harvested and stained with FLICA1 reagent. One experiment representative of three is shown. ^∗^p < 0.05; two-way ANOVA test. (F) IL-1β secretion by WT and *Tmem176b*^*−/−*^ BMDCs treated as in (E) compared with those treated with 10 μM Z-WEHD-FMK 15 min before ATP. One experiment representative of three is shown. ^∗∗^p < 0.01, ^∗∗∗∗^p < 0.0001; two-way ANOVA test. (G and H) Determination of IL-1β (G) and IL-18 (H) by ELISA in culture supernatants from WT, *Tmem176b*^*−/−*^, and *Tmem176b*^*−/−*^*Casp1*^*−/−*^ BMDCs treated as in (E). One experiment representative of two is shown. ^∗^p < 0.05, ^∗∗^p < 0.01, ^∗∗∗∗^p < 0.0001; two-way ANOVA test. (I) Determination of IL-1β in culture supernatants of THP-1-differentiated macrophages expressing GFP or GFP-TMEM176B untreated or treated for 3 h with 0.25 μg/mL LPS and then for 2 h with 2.5 μM Nig. One experiment representative of four is shown. ^∗∗^p < 0.01, ^∗∗∗^p < 0.001; two-way ANOVA test. (J) Calcium determination in WT and *Tmem176b*^*−/−*^ BMDCs treated for 3 h with 0.25 μg/mL LPS and 0.5 mM ATP. Cells were loaded with Ca^2+^-sensitive probe Fura-2. Emission at 340/380 nm was recorded in time-lapse experiments; 0.5 mM ATP was added when indicated by the arrow. Scale bars, 10 μm. (K) Determination of IL-1β in BMDCs exposed to the NLRP3 inflammasome activator ATP as described in (E) in the presence or absence of the intracellular Ca^2+^ chelator BAPTA (100 μM) or DMSO vehicle control. One experiment representative of three is shown. ^∗^p < 0.05; two-way ANOVA test. (L) Determination of IL-1β in BMDCs following inflammasome activation in the presence of control buffer (5 mM) or high K^+^ buffer (120 mM). One experiment representative of three is shown. ^∗^p < 0.05; two-way ANOVA test. (M and N) Determination of IL-1β in BMDCs following inflammasome activation in the presence or absence of the Ca^2+^-activated K^+^ channels blockers iberiotoxin (IbTx) in (M) or hydroxychloroquine (HCQ) in (N). One experiment representative of three is shown in each case. ^∗^p < 0.05, ^∗∗^p < 0.01; two-way ANOVA test. In ELISA experiments, ND stands for not detected. Mean ± SD are shown. See also Figure S1.

To investigate whether TMEM176B regulates inflammasome activation, we injected ATP in wild-type (WT) or *Tmem176b*^*−/−*^ mice (Figure 1A). In this model, neutrophil recruitment to the peritoneal cavity relies on caspase-1/11 activation (Schroeder et al., 2017). We observed that *Tmem176b*^*−/−*^ mice recruited significantly more neutrophils than WT animals. To determine whether increased neutrophil recruitment upon ATP injection was dependent on inflammasome activation, we generated *Tmem176b*^*−/−*^*Casp1*^*−/−*^ double knockout (DKO) mice (Figure S1A). Peritoneal neutrophil recruitment was almost completely inhibited in *Tmem176b*^*−/−*^*Casp1*^*−/−*^ compared with *Tmem176b*^*−/−*^ animals (Figure 1A). ATP-induced neutrophil recruitment in *Tmem176b*^*−/−*^ mice was also interrupted by injection of a caspase-1 inhibitor (Figure S1B). We then stimulated WT and *Tmem176b*^*−/−*^ bone marrow-derived DCs (BMDCs) with the well-established NLRP3 activators ATP and nigericin and determined IL-1β in culture supernatants as a readout of inflammasome activation. We observed that, for both stimuli, *Tmem176b*^*−/−*^ BMDCs secreted significantly higher levels of IL-1β than WT DCs in a dose- and time-dependent manner (Figures 1B and 1C). Similar findings were observed when we stimulated BMDCs with aluminum particles (Figure S1C). Western blot studies confirmed that the mature (cleaved) form of IL-1β was more abundant in culture supernatants from *Tmem176b*^*−/−*^ BMDCs compared with those obtained from WT cells (Figure 1D). Moreover, we observed increased mature caspase-1 in supernatants from *Tmem176b*^*−/−*^ BMDCs compared with WT cells when stimulated with ATP (Figure 1D). Although lower doses (2.5 μM) of nigericin induced expression of mature caspase-1 in culture supernatants from *Tmem176b*^*−/−*^ but not WT BMDCs (Figure 1D), higher doses (5 μM) of this NLRP3 activator induced cleavage of caspase-1 in WT DCs, whereas lipopolysaccharides (LPS) alone did not (Figure S1D). In agreement with this observation, flow cytometry studies using the FLICA1 reagent revealed higher caspase-1 activation in *Tmem176b*^*−/−*^ BMDCs (Figure 1E), suggesting that caspase-1 may contribute to mature IL-1β secretion by *Tmem176b*^*−/−*^ BMDCs. To confirm these findings, we induced inflammasome activation in WT and *Tmem176b*^*−/−*^ BMDCs in the presence or absence of a caspase-1 inhibitor and found that IL-1β secretion was completely inhibited when caspase-1 activation was interrupted (Figure 1F). Moreover, IL-1β secretion was completely abrogated in *Tmem176b*^*−/−*^*Casp1*^*−/−*^ BMDCs (Figure 1G). Thus, increased IL-1β secretion observed as a result of *Tmem176b* deficiency requires intact caspase-1 activity. Moreover, *Tmem176b*^*−/−*^ BMDCs also secreted higher amounts of IL-18 compared with WT cells in a caspase-1-dependent manner (Figure 1H).

We then speculated that TMEM176B overexpression may impair IL-1β secretion in cells in which the inflammasome was activated. To address this issue, THP-1-differentiated macrophages were transfected with TMEM176B/GFP or GFP alone (Figure S1E) and then treated with LPS and nigericin. TMEM176B overexpression impaired IL-1β secretion compared with GFP-expressing cells (Figure 1I); this effect was not associated with increased cell death (Figure S1F). Thus, cation channel TMEM176B inhibits activation of the NLRP3 inflammasome. On the other hand, TMEM176B expression in BMDCs appears to be modulated, at least in part, by inflammasome activation (Figure S1G), suggesting bidirectional regulation of these pathways. Of note, lower TMEM176B expression was not associated with widespread inhibition of inflammatory mediators (Figure S1G) or with increased cell death (Figure S1H) in inflammasome-stimulated BMDCs.

### TMEM176B Inhibits the Inflammasome through the Control of Cytosolic Ca^2+^

TMEM176B is an endophagosomal non-selective monovalent cation channel (Segovia et al., 2014). Because the NLRP3 inflammasome is tightly regulated by cytosolic K^+^ (Muñoz-Planillo et al., 2013) and Ca^2+^ (Murakami et al., 2012) levels, we speculated that TMEM176B may inhibit inflammasome activation through the regulation of ion homeostasis. To address this question, we first determined cytosolic Ca^2+^ levels in WT and *Tmem176b*^*−/−*^ BMDCs stimulated with ATP. BMDCs lacking *Tmem176b* showed greater cytosolic Ca^2+^ as compared with WT BMDCs (Figure 1J). Interestingly, intracellular Ca^2+^ chelation completely blocked IL-1β secretion in WT and *Tmem176b*^*−/−*^ BMDCs (Figure 1K). This effect was also dependent on K^+^ efflux in WT and *Tmem176b*^*−/−*^ BMDCs (Figure 1L).

We recently showed that Ca^2+^-activated K^+^ channels are involved in ATP-triggered inflammasome activation (Schroeder et al., 2017). We therefore inhibited Ca^2+^-activated K^+^ channels in ATP-treated WT and *Tmem176b*^*−/−*^ BMDCs and determined the amounts of IL-1β in culture supernatants. Inhibition of channel function using iberiotoxin or hydroxychloroquine (Schroeder et al., 2017) led to dose-dependent reduction in IL-1β secretion by WT BMDCs. Interestingly, IL-1β secretion by *Tmem176b*^*−/−*^ BMDCs was completely abrogated by both inhibitors at doses that partially inhibited IL-1β secretion in WT BMDCs (Figures 1M and 1N). Thus, heightened inflammasome activation in *Tmem176b*^*−/−*^ BMDCs is highly dependent on Ca^2+^-activated K^+^ channels. These results suggest that TMEM176B impairs ATP-induced cytosolic Ca^2+^ accumulation, preventing Ca^2+^-dependent K^+^ channel-driven inflammasome activation.

### Lack of *Tmem176b* Restrains Tumor Growth in an IL-1β- and Caspase-1-Dependent Manner

**Figure 2:**
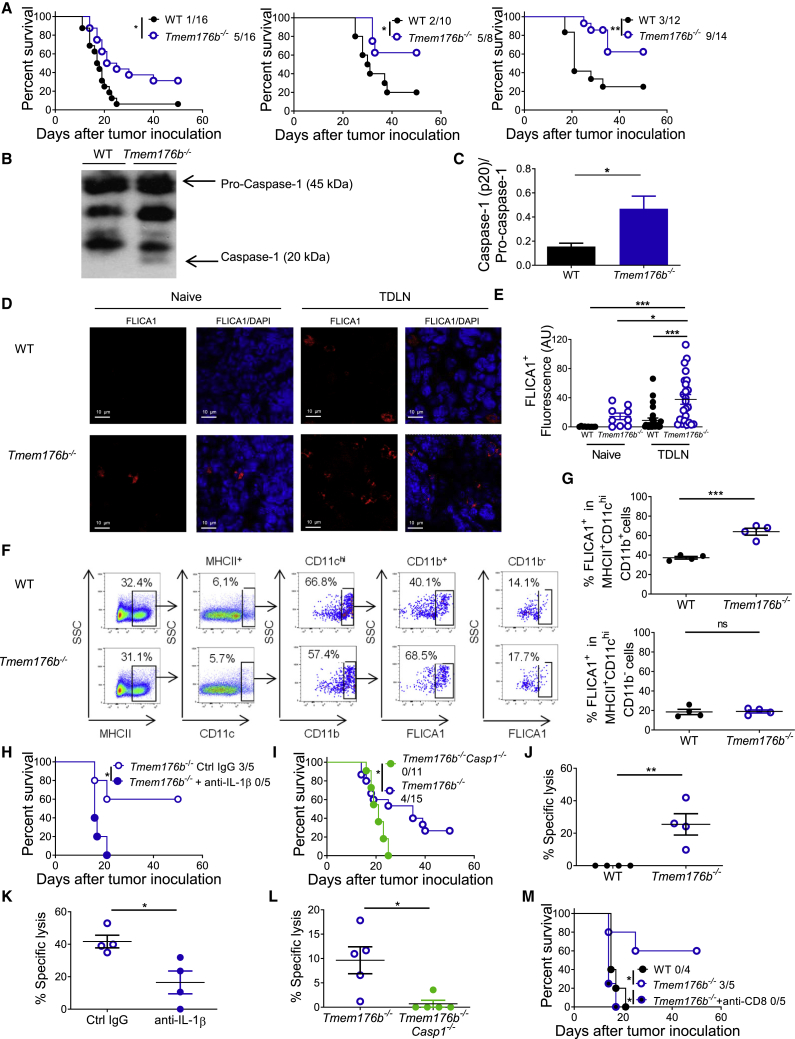
Mice Lacking *Tmem176b* Control Tumor Growth through an IL-1β- and caspase-1-Dependent Manner (A) Survival of WT and *Tmem176b*^*−/−*^ mice injected subcutaneously with MC38 colon cancer cells (1 × 10^6^; left graph), LL2 lung cancer cells (1 × 10^5^; central graph), or EG7 thymic lymphoma cells (1 × 10^6^; right graph). Mice survival was monitored every 3 days. The ratio shows the number of surviving animals/total injected mice from three experiments. ^∗^p < 0.05, ^∗∗^p < 0.01; log rank (Mantel-Cox) test. (B and C) Western blot analysis (B) and semiquantification (C) of pro-caspase-1 and caspase-1 expression in tumor-draining lymph nodes (TDLN) from WT and *Tmem176b*^*−/−*^ mice. At least four animals/group are shown. ^∗^p < 0.05; Student's t test. (D and E) Confocal microscopy (D) and semiquantification (E) of activated caspase-1 expression using the FLICA1 fluorescent probe in TDLN. Scale bars, 10 μm. n = 3 each group. ^∗^p < 0.05, ^∗∗∗^p < 0.001; one-way ANOVA test. (F) Flow cytometry analysis of FLICA1^+^ cells within TDLN. SSC, Side scatter. One experiment representative of two is shown. (G) Evaluation of FLICA1^+^ CD11b^+^ and CD11b^−^ classical DCs (cDCs) in TDLN is shown. ns, not significant; ^∗∗∗^p < 0.001; Student's t test. (H) Survival of *Tmem176b*^*−/−*^ EG7 tumor-bearing mice treated with anti-IL-1β or control immunoglobulin G (IgG) antibodies. The ratio shows the number of surviving animals/total injected mice from one experiment. ^∗^p < 0.05; log rank (Mantel-Cox) test. (I) Survival of untreated *Tmem176b*^*−/−*^ and *Tmem176b*^*−/−*^*Casp1*^*−/−*^ EG7 tumor-bearing mice. The ratio shows the number of surviving animals/total injected mice pooled from three independent experiments. ^∗^p < 0.05; log rank (Mantel-Cox) test. (J) *In vivo* cytotoxicity against OVA-expressing cells in WT and *Tmem176b*^*−/−*^ EG7 tumor-bearing mice. Data from four different animals and one experiment in each group are shown. ^∗∗^p < 0.01; Student's t test. (K) *In vivo* cytotoxicity against OVA-expressing cells in EG7 tumor-bearing *Tmem176b*^*−/−*^ mice treated with anti-IL-1β neutralizing or control IgG antibodies. ^∗^p < 0.05; Student's t test. (L) *In vivo* cytotoxicity against OVA-expressing cells in tumor-bearing *Tmem176b*^*−/−*^ versus *Tmem176b*^*−/−*^*Casp1*^*−/−*^ mice. Data from two experiments are shown. ^∗^p < 0.05; Student's t test. (M) Survival of tumor-bearing WT and *Tmem176b*^*−/−*^ mice left untreated or treated with anti-CD8 depleting antibodies. The ratio depicts the number of surviving animals/total injected mice. Data from one experiment are shown. ^∗^p < 0.05; log rank (Mantel-Cox) test. The genetic background of the animals used was C57BL/6. Mean ± SD are shown. See also Figures S2–S5.

To investigate whether TMEM176B-mediated regulation of inflammasome activation may influence antitumor immunity, we first examined the relevance of TMEM176B expression in human cancer. High stromal TMEM176B expression in colon cancer was associated with significantly lower overall patient survival (Figures S2A and S2B). Moreover, we detected a striking negative correlation between *TMEM176B* and *NLRP3/IL1B* expression from single-cell RNA sequencing analysis in macrophages infiltrating human melanoma (data analyzed from Jerby-Arnon et al., 2018), suggesting a role for this axis in the tumor microenvironment (Figure S2C). Accordingly, *Tmem176b*^*−/−*^ mice inoculated with MC38 (colon), LL/2 (LLC1; lung), or EG7 (thymic lymphoma) cell lines showed higher survival (Figure 2A) and reduced tumor growth (Figure S2D) compared with WT mice. Although TMEM176B is expressed by the three tumor cell lines studied (Figure S2E), immune cells from tumor-bearing *Tmem176b*^*−/−*^ animals did not show enhanced *in vivo* cytotoxicity against WT cells compared with tumor-bearing *Tmem176b*^*+/+*^ mice (Figure S2F), suggesting that tumor-associated TMEM176B is not immunogenic in *Tmem176b*^*−/−*^ hosts.

To investigate the mechanisms underlying TMEM176B contribution to tumor growth, we studied inflammasome activation and found no differences in caspase-1 activation in tumors developed in WT and *Tmem176b*^*−/−*^ mice (Figure S2G). However, we found increased caspase-1 activation in tumor-draining lymph nodes (TDLN) from *Tmem176b*^*−/−*^ mice compared with WT animals (Figures 2B–2E). Moreover, flow cytometry analysis revealed augmented caspase-1 activation in resident CD11c^hi^ MHC II^+^ CD11b^+^ classical DCs (cDCs) in TDLN from *Tmem176b*^*−/−*^ versus WT tumor-bearing mice (Figures 2F, 2G, and S2H). Migratory and resident cDCs were discriminated based on CD11c and MHC II expression (Figure S2H) as described (Laoui et al., 2016). Interestingly, CD11c^hi^ MHC II^+^ CD11b^+^ cDCs expressed considerable amounts of TMEM176B (Crozat et al., 2011) and TDLN contained higher frequency of CD11b^+^ TMEM176B^+^ cells compared with lymph nodes from naive animals (Figure S2I).

Since CD11b^+^ cDCs induce differentiation of Th17 cells (Durai and Murphy, 2016), we speculated that this CD4^+^ T cell subset may augment in TDLN from *Tmem176b*^*−/−*^ mice. We observed increased frequency of TCRβ^+^ CD4^+^ RORγt^+^ cells in TDLN from *Tmem176b*^*−/−*^ animals compared with WT and anti-IL-1β-treated *Tmem176b*^*−/−*^ mice (Figure S2J). Moreover, *in vitro* re-stimulation of TDLN cells with ovalbumin (OVA) showed increased proportion of IL-17^+^ CD4^+^ T cells in *Tmem176b*^*−/−*^ compared with WT mice (Figure S2K), and *in vivo* IL-17A blockade showed a clear trend toward suppression of the antitumor effect in tumor-bearing *Tmem176b*^*−/−*^ mice (Figure S2L). Thus, *Tmem176b* deficiency is associated with an enhanced frequency of functional TCRβ^+^ CD4^+^ RORγt^+^ IL-17^+^ T cells in an inflammasome-dependent manner.

To study whether increased inflammasome activation could be responsible for tumor control in mice lacking *Tmem176b*, we blocked IL-1β and studied EG7 tumor development. Treatment with anti-IL-1β-neutralizing antibodies, but not with control immunoglobulin G, eliminated the antitumor activity displayed by *Tmem176b*^*−/−*^ mice (Figure 2H). This effect was also verified in *Tmem176b*^*−/−*^*Casp1*^*−/−*^ DKO mice (Figure 2I). These results suggest that diminished tumor growth observed in *Tmem176b*^*−/−*^ mice depends on inflammasome activation.

To further examine the cellular effectors involved in tumor growth inhibition in *Tmem176b*^*−/−*^ mice, we analyzed a panel of immunological mediators by qRT-PCR and found no differences between tumors grown in WT or *Tmem176b*^*−/−*^ mice (Figure S3A). Moreover, we did not find significant changes in the percentage or absolute number of infiltrating myeloid, B, NK, NKT, or CD4^+^ T cells between WT and *Tmem176b*^*−/−*^ tumors (Figure S3B). However, the percentage of total CD8^+^ T cells within tumor infiltrates, as well as the absolute number of total and tumor-specific CD8^+^ T cells, was considerably increased in tumors grown in *Tmem176b*^*−/−*^ mice compared with those developed in WT mice (Figures S4A and S4B). Although the absolute number of CD4^+^ CD25^+^ Foxp3^+^ regulatory T (Treg) cells was higher in tumors developed in *Tmem176b*^*−/−*^ versus WT animals, an increased effector T cell (CD8)/Treg (Foxp3) ratio was apparent (Figure S4C). Moreover, tumor-infiltrating CD8^+^ T cells from *Tmem176b*^*−/−*^ mice showed greater proliferation compared with those obtained from WT animals when re-stimulated *in vitro* with OVA MHC I peptide (Figure S4D). Interestingly, we found downregulation of the Treg-related molecules Foxp3, CTLA-4, CCL5, CCL19, and CCL22 in TDLN from *Tmem176b*^*−/−*^ versus WT mice (Figure S3A). Moreover, decreased percentages but not absolute numbers of TCRβ^+^ CD4^+^ Foxp3^+^ Treg cells were observed in TDLN from *Tmem176b*^*−/−*^ versus WT mice, and the CD8/Treg ratio in TDLN was significantly increased in *Tmem176b*^*−/−*^ mice (Figures S5A and B). *In vivo*, MHC I-dependent CD8^+^ T cell-mediated cytotoxicity against OVA-expressing cells was increased in tumor-bearing *Tmem176b*^*−/−*^ compared with WT mice (Figures 2J and S5C). This effect was prevented in *Tmem176b*^*−/−*^ animals treated with anti-IL-1β antibodies (Figure 2K) as well as in *Tmem176b*^*−/−*^*Casp1*^*−/−*^ animals (Figure 2L). Within the tumor microenvironment, CTLs from *Tmem176b*^*−/−*^*Casp1*^*−/−*^ animals expressed lower levels of the degranulation marker CD107a than those from *Tmem176b*^*−/−*^ mice (Figure S5D). Interestingly, depletion of CTLs in *Tmem176b*^*−/−*^ mice using an anti-CD8 antibody increased tumor growth to similar levels as those observed in WT mice (Figure 2M). Thus, *Tmem176b* deletion enhances CTL-mediated tumor control through mechanisms involving the caspase-1/IL-1β pathway. This mechanism is associated with inflammasome-dependent induction of TCRβ^+^ CD4^+^ RORγt^+^ cells. Altogether, these results support a role for TMEM176B as an emerging immune checkpoint that interrupts inflammasome activation and links innate and adaptive antitumor responses.

### Inflammasome Activation Reinforces Immune Checkpoint Blockade Therapies

**Figure 3:**
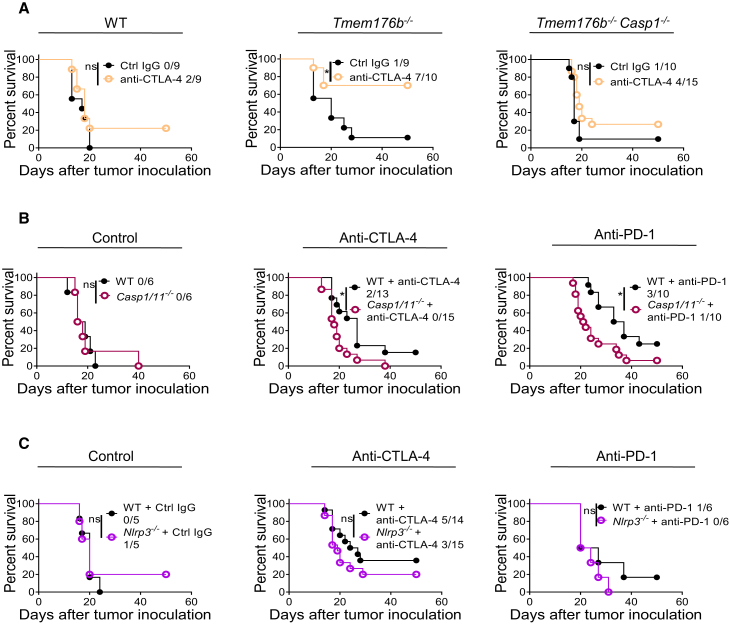
Inflammasome Activation Reinforces Immune Checkpoint Blockade (A) Survival of WT, *Tmem176b*^*−/−*^, and *Tmem176b*^*−/−*^*Casp1*^*−/−*^ mice inoculated with EG7 tumor cells and receiving anti-CTLA-4 or control IgG antibodies. ^∗^p < 0.05; log rank (Mantel-Cox) test. (B and C) Survival of WT and *Casp1/11*^*−/−*^ (B) or *Nlrp3*^*−/−*^ (C) mice inoculated with EG7 tumor cells and injected with control IgG, anti-CTLA-4, or anti-PD-1 antibodies. ns, not significant; ^∗^p < 0.05; log rank (Mantel-Cox) test. Data from three (A and B) or two (C) experiments are shown. The ratio depicts the number of surviving animals/total injected mice.

Given the influence of *Tmem176b* deletion in antitumor immunity, we investigated whether targeting this ion channel might control the efficacy of immune checkpoint blockade. We found increased survival of *Tmem176b*^*−/−*^ compared with WT tumor-bearing mice following treatment with anti-CTLA-4 monoclonal antibody (mAb) (Figure 3A). This effect was dependent on inflammasome activation, as it was abrogated in *Tmem176b*^*−/−*^*Casp1*^*−/−*^ animals (Figure 3A). To investigate this further, we injected anti-CTLA-4 or anti-PD-1 mAb in EG7 tumor-bearing C*asp1/11*^*−/−*^ or *Nlrp3*^*−/−*^ mice. Lack of *Casp1/11* eliminated the antitumor effects triggered by CTLA-4 or PD-1 blockade (Figure 3B). Although the experiments performed in *Nlrp3*^*−/−*^ mice did not reach statistical significance, there was a trend toward lower survival in those mice when treated with anti-CTLA-4 or anti-PD-1 mAb (Figure 3C). Moreover, we found no differences in tumor growth in mice lacking inflammasome components under control conditions (Figures 3B and 3C), in agreement with previous reports (Ghiringhelli et al., 2009). These results highlight the importance of triggering inflammasome activation to improve the efficacy of checkpoint blockade therapies.

### Sensitivity to Immune Checkpoint Blockers Is Associated with an “Inflammasome-Activated” Signature in Cancer Patients

**Figure 4:**
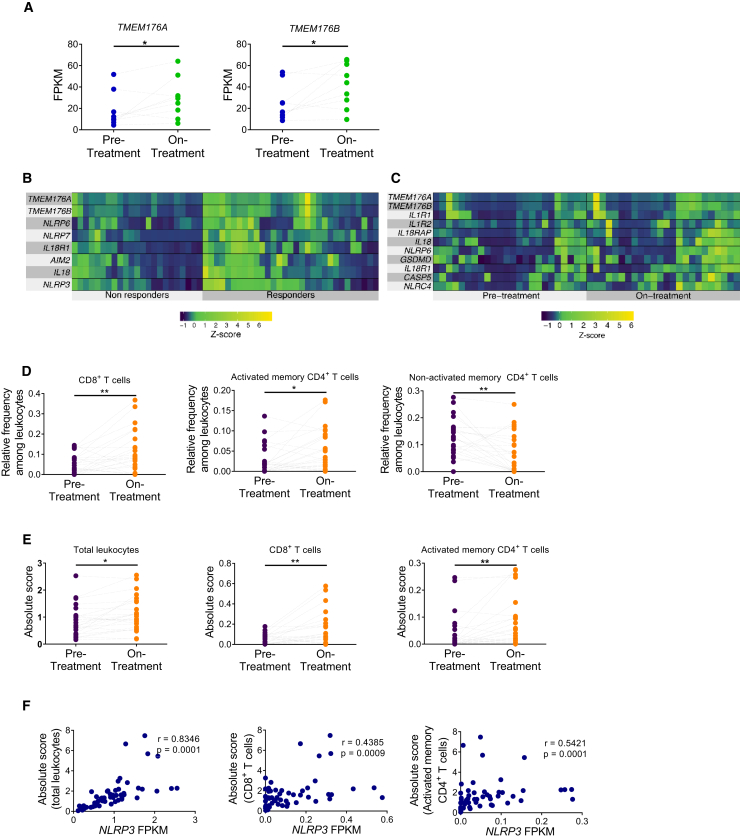
Analysis of the Inflammasome Signature in Tumor Biopsies from Melanoma Patients Treated with Immune Checkpoint Blockers (A) Paired analysis comparing pre-treatment versus on-treatment melanoma biopsies of patients who did not respond to anti-PD-1 therapy and were not treated previously with anti-CTLA-4 antibody (IPI-naive). ^∗^p < 0.05; paired Student's t test. (B) Heatmaps of transcriptome analysis from tumor biopsies of melanoma patients responding (responders) or not (non-responders) to anti-PD-1 therapy. ^∗^p < 0.05; unpaired Student's t test. (C) Paired analysis comparing pre-treatment versus on-treatment melanoma biopsies of patients responding to anti-PD-1 therapy. The indicated inflammasome-related genes were significantly upregulated during therapy. p < 0.05; paired Student's t test. (D and E) Paired study of the relative frequency (D) and absolute number (E) of the indicated cell populations between pre-treatment versus on-treatment tumor biopsies from total patients responding to anti-PD-1 therapy analyzed by the CIBERSORT method. ^∗^p < 0.05, ^∗∗^p < 0.01; paired Student's t test. (F) Association of *NLRP3* expression with the frequency of total leukocytes, CD8^+^ T cells and activated memory CD4^+^ T cells in patients responding to anti-PD-1 therapy. Results show transcriptomics data obtained from tumor biopsies at the on-treatment stage. See also Tables S1–S6.

We then investigated whether inflammasome-related genes might be associated with clinical responses in patients treated with immune checkpoint blockers. First, we analyzed whole-exome sequencing and transcriptomics data from a cohort of melanoma patients treated with immune checkpoint inhibitors (Riaz et al., 2017). These studies focused on pre-treatment and on-treatment tumor biopsies from patients receiving anti-PD-1 mAb after progression from anti-CTLA-4 therapy (IPI-progressing) and patients treated with anti-PD-1 without previous anti-CTLA-4 treatment (IPI-naive). In non-responding patients of the IPI-naive population, only two inflammasome-related genes—*TMEM176A* and *TMEM176B*—were significantly upregulated during treatment compared with pre-treatment (Figure 4A; Table S1). These observations emphasize the role of TMEM176 ionic channels as potential mediators of resistance to checkpoint blockade therapies.

Interestingly, when comparing patients responding or not to anti-PD-1 at the pre-treatment stage, we found no significant differences in inflammasome-related genes in the entire population (Table S2), or in the IPI-naive (Table S3) or IPI-progressed (Table S4) groups. However, eight inflammasome-related genes were significantly upregulated in responders versus non-responders in the entire population during anti-PD-1 treatment (Figure 4B). *TMEM176A* and *TMEM176B* were two of the inflammasome-related genes that were significantly upregulated in patients responding to anti-PD-1, suggesting that they could function as a counter-regulatory mechanism in response to treatment. Similar findings were observed in the IPI-naive population (Table S5). We then performed a paired analysis of tumor biopsies comparing pre-treatment and on anti-PD-1 treatment from responding patients. We found 11 inflammasome-related genes that were significantly upregulated during anti-PD-1 therapy compared with pre-treatment biopsies (Figure 4C). Similar results were found when analyzing the IPI-naive population (Table S6).

We then estimated the diversity of leukocyte populations infiltrating tumors using the CIBERSORT method (Newman et al., 2015). We observed increased relative frequencies of CD8^+^ T cells and activated memory CD4^+^ T cells during anti-PD-1 treatment versus pre-treatment in responders but not in progressors (Figure 4D). Absolute number of leukocytes, CD8^+^ T cells, and activated memory CD4^+^ T cells were also increased (Figure 4E). In patients responding to anti-PD-1 therapy, the total number of leukocytes, as well as the frequency of CD8^+^ T cells and activated memory CD4^+^ T cells, were positively associated with expression of *NLRP3* during ongoing treatment (Figure 4F). These observations reinforce the concept that inflammasome activation controls T cell immunity in patients treated with immune checkpoint blockers.

**Figure 5:**
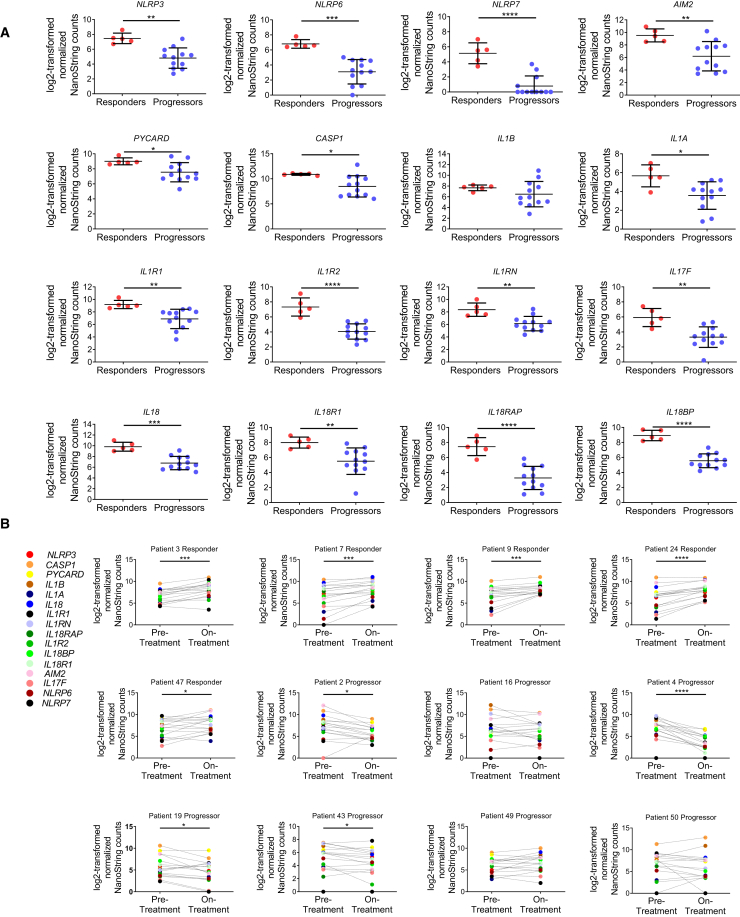
Analysis of the Inflammasome Signature in Tumor Biopsies from Melanoma Patients Treated with Immune Checkpoint Blockers (A) Log2-transformed normalized NanoString counts for the indicated inflammasome-related genes in melanoma tumor biopsies from patients being treated with anti-PD-1 mAb. The results for responding and non-responding patients as defined by Chen et al. (2016). Mean ± SD are shown. ^∗^p < 0.05, ^∗∗^p < 0.01, ^∗∗∗^p < 0.001, ^∗∗∗∗^p < 0.0001; unpaired Student's t test. (B) Paired analysis of the 16 inflammasome-related genes studied in (A) comparing pre-treatment and on-treatment tumor biopsies from melanoma patients responding (n = 5) or not responding (n = 7) to anti-PD-1 therapy. ^∗^p < 0.05, ^∗∗∗^p < 0.001, ^∗∗∗∗^p < 0.0001; paired Student's t test. See also Figures S6 and S7.

To validate further these observations, we analyzed the inflammasome gene expression profile in longitudinal tumor biopsies from melanoma patients treated sequentially with anti-CTLA-4 and anti-PD-1 mAb (Chen et al., 2016). These authors studied gene expression profiling (GEP) via a custom 795-gene panel composed of immune and cancer-related genes which did not include *TMEM176A* and *TMEM176B*. The authors found no significant differences in GEP when comparing responders versus progressors before anti-CTLA-4 or anti-PD-1 therapy. Consistently, we found no significant expression of inflammasome-related genes at these stages (Figures S6A, S6B, S7A, and S7B). These results are in agreement with our findings from the analysis of the Riaz et al. cohort at the pre-treatment stage (Tables S1–S4). However, the authors found 411 genes that were significantly regulated (mostly upregulated) in responders versus progressors following PD-1 blockade. In those patients, 15/16 inflammasome-related genes were significantly upregulated in responders compared with progressors (Figure 5A). We then performed a paired analysis of the 16 inflammasome-related genes in biopsies of 5 responders and 7 progressors comparing gene expression profiles before and during anti-PD-1 therapy. All these patients had progressed to anti-CTLA-4 therapy. Critically, 5/5 patients responding to anti-PD-1 mAb showed a significant upregulation of inflammasome-related genes during anti-PD-1 treatment (Figure 5B). Moreover, 4/7 patients who did not respond to anti-PD-1 therapy significantly downregulated the inflammasome signature during PD-1 blockade (Figure 5B).

Thus, gene expression profiles from biopsies of two independent cohorts of melanoma patients treated with immune checkpoint blockers revealed strong association between inflammasome activation and clinical responses. These findings support the notion that inflammasome activation contributes to antitumor responses triggered by immune checkpoint blockers and highlights the value of an “inflammasome activation” signature as a potential biomarker of response to immune checkpoint blockade.

### Pharmacologic Inhibition of TMEM176B Triggers Inflammasome-Dependent Tumor Control and Improves the Efficacy of Immune Checkpoint Blockers

**Figure 6:**
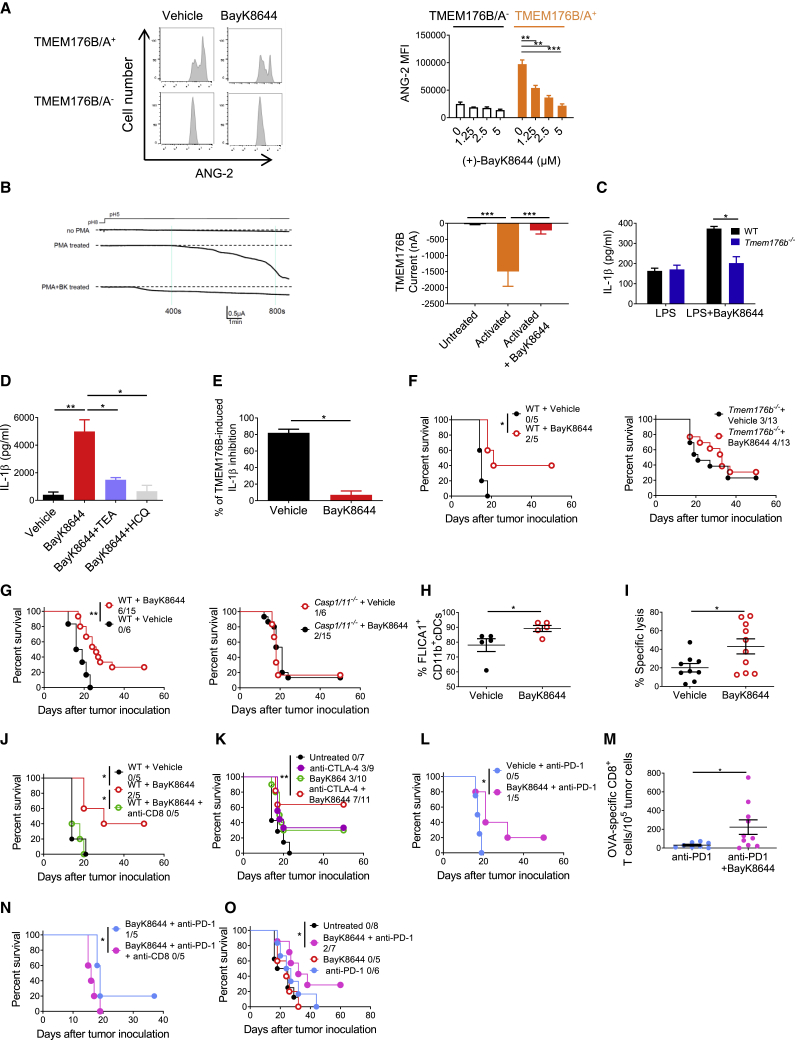
Targeting TMEM176B with BayK8644 Triggers Inflammasome-Dependent Antitumor Immunity (A) TMEM176B activity in CHO-7 cells treated or not with BayK8644. Representative flow cytometry histograms displaying ANG-2 fluorescence at the indicated conditions (left) and quantification of ANG-2 mean fluorescence intensity (MFI) (right). One experiment representative of five is shown. ^∗∗^p < 0.01, ^∗∗∗^p < 0.001; two-way ANOVA test. (B) TMEM176B-dependent conductance assessed in *Xenopus* oocytes following addition of 10 μM (+) BayK8644 to extracellular buffer during phorbol myristate acetate (PMA) stimulation. Representative currents (left) and determination of TMEM176B current at 800 s post-extracellular acidification (right) are shown. ^∗∗∗^p < 0.001; one-way ANOVA test. (C) Determination of IL-1β in culture supernatants from WT and *Tmem176b*^*−/−*^ BMDCs primed for 3 h with LPS and then treated or not with 2.5 μM BayK8644. One experiment representative of three is shown. ^∗^p < 0.05; two-way ANOVA test. (D) Determination of IL-1β in culture supernatants from WT BMDCs primed with LPS and then treated with 10 μM BayK8644 alone or in combination with tetraethylammonium (TEA) (2 mM) or HCQ (10 μM). One experiment representative of three is shown. ^∗^p < 0.05, ^∗∗^p < 0.01; one-way ANOVA test. (E) Determination of IL-1β in culture supernatants from THP-1-differentiated macrophages transfected with GFP or TMEM176B/GFP-coding plasmids and then treated or not with LPS plus nigericine (LPS/Nig) in the presence of ethanol (vehicle) or 5 μM BayK8644. To calculate the extent of TMEM176B-dependent inhibition, IL-1β levels (pg/mL) were incorporated to the formula: [GFP/LPS/Nig – GFP untreated] – TMEM176B/LPS/Nig × 100. One experiment representative of three is shown. ^∗^p < 0.05; Student's t test. (F and G) Survival of WT (F and G) and *Tmem176b*^*−/−*^ (F) or *Casp1/11*^*−/−*^ (G) mice inoculated with EG7 tumor cells and treated with 1 mg/kg BayK8644 i.p. on days 2–15 after tumor cell injection. ^∗^p < 0.05, ^∗∗^p < 0.01; log rank (Mantel-Cox) test. (H) Caspase-1 activation in TDLN from WT mice inoculated with EG7 tumor cells and then treated or not with 1 mg/kg BayK8644 on days 2–13. TDLN were resected 14 days after tumor injection and caspase-1 activation was studied by flow cytometry using the FLICA1 reagent. ^∗^p < 0.05; Student's t test. (I) *In vivo* cytotoxicity against OVA-expressing cells in WT mice inoculated with EG7 tumor cells treated or not with BayK8644 as in (F). At day 15, *in vivo* cytotoxicity was determined. ^∗^p < 0.05; Student's t test. (J) Survival of tumor (EG7)-bearing WT mice treated with BayK8644 or vehicle control, receiving or not anti-CD8 depleting antibody. ns, not significant. WT + Vehicle versus WT + BayK8644: ^∗^p < 0.05; WT + BayK8644 versus WT + BayK8644 + anti-CD8: ^∗^p < 0.05; WT + Vehicle versus WT + BayK8644 + anti-CD8: ns; log rank (Mantel-Cox) test. (K) Survival of tumor (EG7)-bearing WT mice treated or not with BayK8644, anti-CTLA-4 mAb, or BayK8644 plus anti-CTLA-4 mAb. ns, not significant. Untreated versus BayK8644 + anti-CTLA-4: ^∗∗^p < 0.01; BayK8644 versus BayK8644 + anti-CTLA-4: ns; anti-CTLA-4 versus BayK8644 + anti-CTLA-4: ns; untreated versus anti-CTLA-4: ns; untreated versus BayK8644: ns; log rank (Mantel-Cox) test. (L) Survival of tumor (EG7)-bearing WT mice treated or not with 250 μg anti-PD-1 mAb at days 6, 9, and 12 after tumor inoculation. BayK8644 was injected every day since day 9 (when all mice had established tumors) until day 21. ^∗^p < 0.05; log rank (Mantel-Cox) test. (M) Frequency of OVA-specific CD8^+^ T cells as determined by flow cytometry using fluorescent MHC pentamers in EG7 tumor suspensions from WT mice treated with anti-PD-1 alone or anti-PD-1 + BayK8644 in a therapeutic protocol as in (L). ^∗^p < 0.05; unpaired Student's t test. (N) Survival of tumor (EG7)-bearing WT mice treated or not with BayK8644 plus anti-PD-1 mAb in the absence or presence of anti-CD8 depleting mAb. ^∗^p < 0.05; log rank (Mantel-Cox) test. (O) Survival of WT mice inoculated with 5,555 melanoma cells and left untreated or treated either with anti-PD-1 mAb (days 6, 9, and 12), BayK8644 (days 9–21), or both. All animals had established tumors when BayK8644 treatment was started. ns, not significant. Untreated versus BayK8644 + anti-PD-1: ^∗^p < 0.05; BayK8644 versus BayK8644 + anti-PD-1: ns; anti-PD-1 versus BayK8644 + anti-PD-1: ns; untreated versus anti-PD-1: ns; untreated versus BayK8644: ns; log rank (Mantel-Cox) test. In (F), (G), and (J–O) the ratio represents the number of surviving mice/total injected mice. For these experiments we used C57BL/6 mice. Mean ± SD are shown. See also Figure S8.

To identify compounds capable of improving the efficacy of immune checkpoint blockers by inhibiting TMEM176B-dependent ion flux and triggering inflammasome activation, we set up an *in vitro* assay. In brief, CHO-7 cells were transfected with TMEM176B- and TMEM176A-mCherry. Cells were then loaded with the Na^+^-sensitive fluorescent dye Asante NaTRIUM Green 2 (ANG-2). We observed increased ANG-2 mean fluorescence intensity in mCherry^+^ compared with mCherry^−^ cells (Figure 6A). We then screened a library of compounds known to modulate ion channel activity (Data S1). We found that both enantiomers of BayK8644 potently inhibited TMEM176B-A-dependent Na^+^ influx, while they minimally affected TMEM176B-A-negative cells (Figures 6A, 6B, and S8A), thus prompting the study of these compounds. Whereas (+) BayK8644 is known to inhibit L-type voltage-dependent Ca^2+^ channels, the (−) stereoisomer activates those channels (Hamilton et al., 1987). Since both isomers inhibit TMEM176B/A activity, it is unlikely that our observations could be explained by their effects on Na^+^ influx through the modulation of Ca^2+^ channels. In electrophysiology studies using TMEM176B-overexpressing *Xenopus* oocytes, (+) BayK8644 completely inhibited TMEM176B-dependent current (Figure 6B). Therefore, we focused on the (+) isomer for functional experiments.

We found that BayK8644 induced IL-1β secretion and caspase-1 activation in LPS-primed WT but not in *Tmem176b*^*−/−*^ BMDCs (Figures 6C and S8B–S8D). Interestingly, BayK8644-induced IL-1β secretion was inhibited by the Ca^2+^-activated K^+^ channel (KCa) inhibitors tetraethylammonium and hydroxychloroquine (Figure 6D). Thus, BayK8644 treatment on WT BMDCs phenocopied *Tmem176b* deficiency. In THP-1-differentiated macrophages, TMEM176B-dependent inhibition of IL-1β secretion was prevented when these cells were treated with BayK8644 (Figure 6E). These results suggest that BayK8644 triggers inflammasome activation through inhibition of TMEM176B.

We then explored whether BayK8644 treatment may control tumor growth *in vivo*. Administration of BayK8644 significantly increased survival of tumor-bearing WT but not *Tmem176b*^*−/−*^ mice (Figure 6F) compared with injection of vehicle control. Of note, *in vitro* treatment of EG7 thymic lymphoma cells with BayK8644 did not induce apoptosis at similar doses as those detected in plasma after intraperitoneal injection (Figure S8E). To explore whether BayK8644 recapitulated the effects in tumor growth control observed in untreated *Tmem176b*^*−/−*^ mice, we evaluated its influence on inflammasome activation by disrupting important components of this pathway. We found that BayK8644 significantly improved survival of WT but not *Casp1/11*^*−/−*^ tumor-bearing mice (Figure 6G). Consistent with this observation, BayK8644 increased the frequency of CD11b^+^ cDCs expressing active caspase-1 in TDLN (Figure 6H). Moreover, BayK8644-induced tumor control was mediated by CD8^+^ T cells as it increased CD8^+^ T cell-dependent tumor cytotoxicity *in vivo* (Figure 6I), and depletion of CD8^+^ T cells completely abolished the antitumor effect of this inhibitor (Figures 6J and S8F). Thus, BayK8644 restrains EG7 tumor growth in a TMEM176B-, caspase-1/11-, and CD8^+^ T cell-dependent manner, phenocopying *Tmem176b*^*−/−*^ mice. Moreover, BayK8644 significantly impaired growth of CT26 colon cancer cells in BALB/c mice (Figures S8G and S8H). Thus, BayK8644 emerges as an immunotherapeutic agent that limits tumor growth by licensing inflammasome activation.

Finally, we evaluated whether BayK8644 administration may enhance the antitumor activity of immune checkpoint blockers. Compared with mice receiving monotherapy, administration of BayK8644 in combination with anti-CTLA-4 mAb significantly improved survival of EG7 tumor-bearing mice (Figure 6K). Moreover, therapeutic administration of BayK8644 in mice with established tumors significantly improved the antitumoral effect of anti-PD-1 treatment (Figure 6L), whereas BayK8644 monotherapy was not effective in this therapeutic protocol (data not shown). Interestingly, combination of anti-PD-1 with BayK8644 was associated with an increased absolute number and percentage of TCRβ^+^ CD4^+^ RORγt^+^ T cells in TDLN (Figure S8I) and increased frequency of tumor-specific CD8^+^ T cells within the tumor microenvironment (Figure 6M) compared with anti-PD-1 monotherapy. Depletion of CD8^+^ T cells in mice treated with anti-PD-1 plus BayK8644 abrogated antitumor immunity (Figure 6N). This observation might be explained by concomitant CTL-mediated mechanisms required for both the antitumor activity of BayK8644 (Figures 6J and S8F) and anti-PD-1 mAb (Sharma and Allison, 2015). Thus, as expected, combination treatment strongly relies on the CD8^+^ T cell compartment. Furthermore, BayK8644 significantly enhanced the antitumoral effect of anti-PD-1 therapy in mice bearing 5555 melanoma (Figure 6O), whereas this effect was apparent in LL/2 lung cancer (Figures S8J and S8K) and MC38 colon cancer (Figures S8L and S8M) models, albeit not reaching statistical significance. Moreover, whereas BayK8644 reinforced the antitumor effects of anti-PD-1 treatment in mouse melanoma, it did not enhance tumor growth inhibition induced by anti-CTLA-4 and anti-PD-1 combination therapy, at least in this model (Figure S8O). Given the pharmacologic impact of channel inhibitors in cardiomyocyte function, we finally examined whether BayK8644 may lead to acute cardiac toxicity. Notably, BayK8644 treatment was not associated with electrocardiographic nor echocardiographic alterations 30 min after intravenous injection compared with mice treated with vehicle control (Tables 1 and 2). Thus, pharmacological inhibition of TMEM176B represents a potential therapeutic approach to unleash inflammasome activation, leading to potentiation of CD8^+^ T cell-dependent antitumor immunity.

**Table 1:** Effect of BayK8644 on Electrocardiographic Parameters

	RR	P wave	PR	QRS	QT	QTc
Control^a^	150 ± 18^b^	16.0 ± 0.0	32.0 ± 2.0	13.3 ± 1.2	53.3 ± 4.1	43.6 ± 0.8
Vehicle	143 ± 9	14.7 ± 0.7	32.0 ± 1.5	13.7 ± 1.8	56.0 ± 2.5	46.8 ± 0.8
Control	130 ± 2	15.2 ± 1.2	32.8 ± 1.9	11.0 ± 0.4	51.2 ± 2.7	45.1 ± 2.7
BayK8644	120 ± 5	15.4 ± 1.2	32.6 ± 1.8	11.2 ± 0.6	54.4 ± 1.9	49.7 ± 0.8

a Values before injection of vehicle control or BayK8644.

b Mean ± SD are expressed in ms.

**Table 2:** Effect of BayK8644 on Echocardiographic Parameters

	Vehicle	BayK8644
Cardiac frequency (bpm)	457 ± 50	515 ± 35
Left ventricular telediastolic wall thicknesses (mm)	1.0 ± 0.0	1.1 ± 0.1
Left ventricular telediastolic diameter (mm)	3.2 ± 0.2	2.8 ± 0.2
Left ventricular ejection fraction (%)	82 ± 1	93 ± 2
E/A ratio	1.7 ± 0.1	1.5 ± 0.1
Isovolumic relaxation time (ms)	15.0 ± 0.0	18.8 ± 0.2
E-wave deceleration time (ms)	37.7 ± 1.8	33.4 ± 2.5

Mean ± SD are expressed.

## Discussion

In this study, we demonstrate a central role of the inflammasome in reinforcing CD8^+^ T cell-dependent antitumor immunity and enhancing the efficacy of checkpoint blockade therapies. In particular, we demonstrate a key role of TMEM176B as negative regulator of inflammasome activation. Whereas most immunotherapeutic modalities have focused on drugs targeting adaptive components of the immune system, innate immune pathways may represent additional anticancer targets (Woo et al., 2014).

Although recently proposed to play a role in immunotherapy (Mangan et al., 2018), the direct contribution of IL-1β/IL-18 inflammasomes to antitumor immunity and adaptive checkpoint blockade remains elusive (Karki et al., 2017). Here we identify a therapeutic strategy that reinforces antitumor responses by targeting TMEM176B and promoting inflammasome disinhibition. Our results suggest that inflammasome activation plays a central role in antitumor immunity triggered by anti-CTLA-4 and anti-PD-1 mAb. Although experiments in *Nlrp3*^*−/−*^ animals did not reach statistical significance, studies in animals lacking the downstream effectors *Casp1/11* did. These observations suggest that different inflammasomes could mediate the antitumoral effect triggered by anti-CTLA-4 and anti-PD-1 therapies. Accordingly, analysis of anti-PD-1-treated melanoma patients suggested that NLRP6, NLRP7, AIM2, and NLRC4 inflammasomes might contribute to antitumor responses unleashed by checkpoint blockers. Although the requirement of caspase-1 autoproteolysis can differ among different inflammasomes (Broz et al., 2010), and caspase-1 may cleave other proteins than IL-1β and IL-18 (Sokolovska et al., 2013), to our knowledge, caspase-1/11 activation mostly depends on inflammasomes. Thus, it is unlikely that observations in *Casp1/11*^*−/−*^ and *Tmem176b*^*−/−*^*Casp1*^*−/−*^ mice could involve inflammasome-independent mechanisms.

Our results suggest that TMEM176B might be a predictive marker of response to anti-PD-1 therapy. In addition, TMEM176B expression in the tumor stroma was associated with poor survival in colorectal cancer patients, suggesting a potential role of this ion channel as a prognostic factor. Interestingly, TMEM176B was associated with diminished *NLRP3* and *IL1B* expression in macrophages infiltrating human melanoma, suggesting that this ion channel may function as an innate checkpoint signal that hinders immune responses in the tumor microenvironment. However, our results in experimental models support a key role for TMEM176B in the modulation of inflammasome activation mostly in TDLN during the induction phase of antitumor responses. Thus, TMEM176B-dependent immune inhibitory mechanisms may operate within the tumor microenvironment and TDLN.

Sustained K^+^ efflux through the voltage-gated (K_v_1.3) or Ca^2+^-activated (KCa3.1) K^+^ channels has been shown to reinvigorate tumor-infiltrating T cells (Eil et al., 2016). The results presented here suggest that Ca^2+^-induced K^+^ efflux in DCs may promote antitumor immunity by triggering inflammasome activation, a process that is repressed by TMEM176B.

In conclusion, our study links inflammasome activation to antitumor responses triggered by immune checkpoint blockers, highlighting a central role for TMEM176B, an ion channel expressed on myeloid cells, in repression of T cell-dependent immunity. Further efforts should be aimed at evaluating the clinical efficacy and safety of inflammasome disinhibition in the treatment of cancer patients, particularly those resistant to current immunotherapies.

## STAR★Methods

### Key Resources Table

**Table:** 

REAGENT or RESOURCE	SOURCE	IDENTIFIER
**Antibodies**		

Goat polyclonal anti-IL-1β	R&D Systems	Cat# AF-401-NA RRID:AB_416684
Control goat IgG	R&D Systems	Cat# AB-108-C RRID:AB_354267
Goat anti-mouse IL-17 polyclonal antibody	R&D Systems	Cat# AF-421-NA RRID:AB_354487
Mouse IgG2b anti-CTLA-4	BioXCell	Cat# BE0164 RRID:AB_10949609
Mouse IgG2b isotype control	BioXCell	Cat# BE0086 RRID:AB_1107791
Rat IgG2a anti-PD-1	BioXCell	Cat# BE0146 RRID:AB_10949053
Rat IgG2a isotype control	BioXCell	Cat# BE0089 RRID:AB_1107769
Anti-CD8a depleting antibody YTS 169.4	BioXCell	Cat# BE0017 RRID:AB_10950145
TCRVb12 (MR11-1)	BD	Cat# 553300 RRID:AB_394768
TCRβ (H57-597)	BD	Cat# 553174 RRID:AB_398534
TCRγδ (GL3)	BD	Cat# 553178 RRID:AB_394689
CD27 (LG.3A10)	BD	Cat# 560691 RRID:AB_1727455
CD49b (DX5)	BD	Cat# 553856 RRID:AB_395092
NK1.1 (PK136)	BD	Cat# 557391 RRID:AB_396674
CD4 (RM4-5)	BD	Cat# 558107 RRID:AB_397030
CD8α (53-6,7)	BD	Cat# 552877 RRID:AB_394506
CD11b (M1/70)	BD	Cat# 553312 RRID:AB_398535
CD11c (HL3)	BD	Cat# 557401 RRID:AB_396684
CD19 (1D3)	BD	Cat# 557398 RRID:AB_396681
CD80 (16-10A1)	BD	Cat# 553769 RRID:AB_395039
CD86 (GL1)	BD	Cat# 553690 RRID:AB_394992
CD107a (1D4B)	BD	Cat# 558661 RRID:AB_1645247
IAb (AF6-120.1)	BD	Cat# 553551 RRID:AB_394918
Ly6C (AL-21)	BD	Cat# 553104 RRID:AB_394628
Ly6G (1A8)	BD	Cat# 551461 RRID:AB_394208
Foxp3 (FJK-16s)	eBiosciences	Cat# 17-5773-80 RRID:AB_469456
Rat IgG2a isotype control (eBR2a)	eBiosciences	25-4321-81 RRID:AB_470199
Anti-caspase-1 (p20) (mouse)	Adipogen	Cat# AG-20B-0042 RRID:AB_2490248
Anti-IL-1β antibody (H-153)	Santa Cruz Biotechnologies	Cat# sc-7884 RRID:AB_2124476
RORγt (AFKJS-9)	eBiosciences	Cat# 17-6988 RRID:AB_1633425
TMEM176B Polyclonal Antibody	Proteintech	Cat# 19825-1-AP RRID:AB_10638313
Anti-LR8 (TMEM176B) antibody	Abcam	Cat# ab103929 RRID:AB_10712259

**Biological Samples**		

Colon cancer tissue array 90 tumor cases with survival information.	US Biomax, Inc	Cat# HCol-Ade180Sur-04

**Chemicals, Peptides, and Recombinant Proteins**		

TRIzol Reagent.	Invitrogen	Cat#15596026
M-MLV Reverse Transcriptase.	Invitrogen	Cat# 28025013
Random primers.	Invitrogen	Cat#48190011
Fast SYBR Green Master Mix.	Applied Biosystems.	Cat#4385612
Lipopolysaccharides from *Escherichia coli* 0111:B4	Sigma	Cat# L4391
Nigericin	Sigma	Cat# N7143; CAS:28643-80-3
ATP	Sigma	Cat# 10127531001 CAS: 51963-61-2
Iberiotoxin	Sigma	Cat# I5904
Hydroxychloroquine	Sigma	Cat# H0915 CAS: 747-36-4
TEA	Sigma	Cat# T2265 CAS: 56-34-8
(-) BayK8644	Sigma	Cat# B-133 CAS: 98625-26-4
Capsaicin	Sigma	Cat# M2028 CAS: 404-86-4
1-(1-Adamantyl)ethylamine hydrochloride	Sigma	Cat# 390593 CAS: 1501-84-4
CGP-37157	Sigma	Cat# C8874 CAS: 75450-34-9
Terfenadine	Sigma	Cat# T9652 CAS: 50679-08-8
Nilvadipine	Sigma	Cat# SML0945 CAS: 75530-68-6
TRAM-34	Sigma	Cat# T6700 CAS: 289905-88-0
Picrotoxin	Sigma	Cat# P1675 CAS: 124-87-8
Clotrimazole	Sigma	Cat# C6019 CAS: 23593-75-1
Caspase-1 inhibitor II (Ac-YVAD-CMK)	Santa Cruz Biotechnology	Cat# sc-300323 CAS: 178603-78-6
(+)-BayK8644	Santa Cruz Biotechnology	Cat# sc-364594 CAS: 98791-67-4
Lipofectamine 2000	Thermo Fisher	Cat# 11668027
Fura-2	Thermo Fisher	Cat# F1201 CAS: 108964-32-5
DDAO-SE	Thermo Fisher	Cat# C34564
Asante NaTRIUM Green-*2* AM	Abcam	Cat# Ab142802
FLICA 660 Caspase-1 Assay (FLICA1)	Immunochemistry	Cat# 9122
SCREEN-WELL® Ion Channel ligand library	Enzo Life Sciences	Cat# BML-2805

**Critical Commercial Assays**		

Mouse IL-1β ELISA	Biolegend	Cat# 432603
Human IL-1β ELISA	BD Bioscience	Cat# 557953
Mouse IL-18 ELISA kit	MBL	Cat# 7625
Amaxa Cell Line Nucleofector Kit V	Lonza	Cat# VCA-1003
EnVision+ System- HRP Labelled Polymer	Dako	Cat# K4002

**Deposited Data**		

Bulk RNA expression data Riaz cohort	(Riaz et al., 2017)	https://github.com/riazn/bms038_analysis/tree/master/data
Normalized single cell expression data.	(Jerby-Arnon et al., 2018)	GEO: GSE115978
Mendeley dataset		https://data.mendeley.com/datasets/publish-confirmation/gvj6fc2b8v/1
Normalized NanoString nCounter data	(Chen et al., 2016)	

**Experimental Models: Cell Lines**		

E.G7-OVA	ATCC	Cat# CRL-2113 RRID:CVCL_3505
MC38	Kerafast	Cat# ENH2040 RRID:CVCL_B288
THP-1	ATCC	Cat# TIB-202 RRID:CVCL_0006
CHO-K1	ATCC	Cat# CCL-61 RRID:CVCL_0214
LL/2 (LLC1)	ATCC	Cat# CRL-1642 RRID:CVCL_4358
CT26.WT	ATCC	Cat# CRL-2638 RRID:CVCL_7256
Melanoma 5555	Richard Marais' lab	N/A

**Experimental Models: Organisms/Strains**		

Mouse: C57BL/6J	Institut Pasteur Montevideo	N/A
Mouse: Balb/C	Institut Pasteur Montevideo	N/A
Mouse: *Tmem176b-1*^*-/-*^	Cristina Cuturi’s lab	N/A
Mouse: *Tmem176b*^*+/+-*^	Cristina Cuturi’s lab	N/A
Mouse: *Tmem176b*^*-/-*^*Casp1*^*-/-*^	This paper	N/A
Mouse: *Nlrp3*^*-/-*^ (B6.129S6-*Nlrp3*^*tm1Bhk*^/J	The Jackson Laboratory	Cat# 021302 RRID:IMSR_JAX:021302
Mouse: *Casp1/11*^*-/-*^ (B6N.129S2-*Casp1*^*tm1Flv*^/J)	The Jackson Laboratory	Cat# 016621 RRID:IMSR_JAX:016621
C57BL/6J	The Jackson Laboratory	Cat# 000664 RRID:IMSR_JAX:000664
C57BL/6NJ	The Jackson Laboratory	Cat# 005304 RRID:IMSR_JAX:005304

**Oligonucleotides**		

Primers for RORγt (mRNA) see Table S7	This paper	N/A
Primers for *Il17a*, see Table S7	This paper	N/A
Primers for *Foxp3*, see Table S7	This paper	N/A
Primers for *Tgfb1*, see Table S7	This paper	N/A
Primers for *Il10*, see Table S7	This paper	N/A
Primers for *Ifng*, see Table S7	This paper	N/A
Primers for *Tnfa*, see Table S7	This paper	N/A
Primers for *Ctla4*, see Table S7	This paper	N/A
Primers for *Ccl22*, see Table S7	This paper	N/A
Primers for *Ccl5*, see Table S7	This paper	N/A
Primers for *Il12*, see Table S7	This paper	N/A
Primers for *Il4*, see Table S7	This paper	N/A
Primers for *Gata3*, see Table S7	This paper	N/A
Primers for *Tbx21*, see Table S7	This paper	N/A
Primers for *Cebpb*, see Table S7	This paper	N/A
Primers for *Ccl19*, see Table S7	This paper	N/A
Primers for *Il6*, see Table S7	This paper	N/A
Primers for *Fas*, see Table S7	This paper	N/A
Primers for *Pdl1*, see Table S7	This paper	N/A
Primers for *Tmem176b*, see Table S7	This paper	N/A
Primers for *Gapdh*, see Table S7	This paper	N/A

**Recombinant DNA**		

pcDNA3.1-*rTmem176b*-GFP	Cédric Louvet	N/A
pcDNA3.1GFP	José Badano	N/A
pSecTag2B-PS-*Tmem176b*-V5His	Cédric Louvet	N/A
pSecTag2b-PS-*rTmem176b*-2mcherry	Cédric Louvet	N/A

**Software and Algorithms**		

FlowJo vX.0.7	Flowjo, LLC	N/A
GraphPad Prism 6	GraphPad Software, Inc.	N/A
Rmagic 1.3.0	(Van Dijk D et al., 2018)	https://github.com/KrishnaswamyLab/MAGIC
CIBERSORT	(Newman et al., 2015)	https://cibersort.stanford.edu

### Contact for Reagent and Resource Sharing

Further information and requests for resources and reagents should be directed to and will be fulfilled by the Lead Contact, Marcelo Hill (mhill@pasteur.edu.uy).

### Experimental Models and Subject Details

#### Animals

Six-to-ten weeks old male or female C57BL/6 or BALB/c mice were used (Jackson Lab; Bar Harbor, ME) and bred for up to 20 generations at the Institut Pasteur Montevideo or at the Institute of Biology and Experimental Medicine (IBYME), Buenos Aires. All experiments were performed according to local regulation and approved by the Institut Pasteur de Montevideo and by the Institutional Committee for Care and Use of Laboratory Animals (CICUAL) at IBYME.

*Tmem176b*^*-/-*^ mice were generated in the 129/SvJ strain and heterozygous mice were backcrossed for 10 generations onto the C57BL/6 background (Janvier, Saint Berthevin, France) as reported (Segovia et al., 2014). *Nlrp3*^*-/-*^ (B6.129S6-*Nlrp3*^*tm1Bhk*^/J; 021302) and *Casp1/11*^*-/-*^ (B6N.129S2-*Casp1*^*tm1Flv*^/J; 016621) were from Jackson Laboratory. *Nlrp3*^*-/-*^ animals were compared to 000664 C57BL/6J, and *Casp1/11*^*-/-*^ mice to 005304 C57BL/6NJ. *Tmem176b*^*-/-*^*Casp1*^*-/-*^ mice were generated by microinjecting Crispr/Cas9 targeting *Casp1* in *Tmem176b*^*-/-*^ embryos. F1 animals were genotyped and heterozygous mice were crossed to generate F2 homozygous *Tmem176b*^*-/-*^*Casp1*^*-/-*^ animals. *Casp1* deficiency was confirmed by Western blot (Figure S1). All animal strains including *Tmem176b*^*-/-*^, *Tmem176b*^*+/+*^ *WT* (issued from littermate controls), C57BL/6J, *Nlrp3*^*-/-*^, C57BL/JN, *Casp1/11*^*-/-*^ and *Tmem176b*^*-/-*^*Casp1*^*-/-*^ were bred at a specific pathogen-free animal facility (Institut Pasteur, Montevideo).

#### Cell Lines

EG7 (expressing OVA antigen), LL2, CT26, THP-1 and CHO-K1 cell lines were purchased from ATCC (Manassas, VA). MC38 cells were from Kerafast (Boston, MA). The 5555 melanoma cell lines were kindly provided by R. Marais (Cancer Research UK, Manchester) and cultured as described (Hirata et al., 2015).

#### Tumor Models and Treatments

C57BL/6 mice were injected s.c with 1 x 10^6^ MC38 colon cancer cells, 1 x 10^5^ LL2 lung cancer cells, 2.5 x 10^5^ 5555 melanoma cells or 1 x 10^6^ EG7 thymic lymphoma cells. BALB/C animals were injected with 1 x 10^5^ CT26 colon cancer cells. Injection was performed alternating one WT and one *Tmem176b*^*-/-*^ mouse until completing both groups. In treated animals, alternation was done between drug- and vehicle-treated animals. Tumor growth was measured manually every 2-3 days with a caliper. The two major diameters were taken. Mice were sacrificed when one of the diameters reached 2 cm. In experiments where anti-IL-1β, anti-IL-17A or control IgG were used, 4 μg antibody was injected i.p 7 days after inoculation of tumor cells. Injections were repeated every five days until day 27 post-injection or euthanasia. For depletion of CD8^+^ T cells, 100 μg YTS 169.4 antibody was injected every three days starting from the day before tumor inoculation. Depletion was confirmed in the spleen by flow cytometry. For administration of anti-CTLA-4 mAb or control IgG, 100 μg antibody was given i.p starting from day 6 after tumor inoculation. Injections were repeated every three days until day 12. Anti-PD-1 mAb or control IgG was injected (250 μg i.p) starting from day 6 and every three days until day 12. BayK8644 or vehicle control (ethanol) was given i.p at 1 mg/kg since day 3 until day 15 after tumor inoculation. In animals treated with BayK8644 and anti-CTLA-4 mAb, BayK8644 was injected at days 3-15 every day and anti-CTLA-4 at days 6, 9 and 12 after tumor inoculation. In mice treated with BayK8644 and anti-PD-1 mAb, treatment with the former started at day 9 and repeated every day until day 21 after tumor inoculation. Anti-PD-1 treatment started at day six after tumor inoculation and repeated every three days until day 12.

#### *In Vivo* Inflammasome Activation

C57BL/6 animals were injected i.p with 20 mg/kg ATP. Four hr later, peritoneal lavage was performed using 5 ml PBS. Peritoneal cells were centrifuged and stained with anti-CD11b, anti-Ly6C and anti-Ly6G antibodies. Cells were analyzed by flow cytometry. The percentage of Ly6C^int^ Ly6G^hi^ cells within the CD11b^+^ cell compartment (neutrophils) was determined. The absolute number of neutrophils was calculated for each condition.

#### *In Vitro* Inflammasome Activation

Bone marrow-derived DCs (BMDCs) were differentiated by culturing bone marrow cells for 8 days in the presence of 0.4 ng/ml GM-CSF. At day 8, adherent cells were >95% CD11c^+^CD11b^+^MHC II^int^. Cells were stimulated for 3 hr with 0.25 μg/ml LPS, washed and treated with the indicated doses of ATP or nigericin. The presence of IL-1β was assessed in culture supernatants by ELISA (Biolegend, 432603). To determine Caspase-1 activation, BMDCs were stained with FLICA1 45 min after ATP or nigericin stimulation and analyzed by flow cytometry. For Western blot, culture supernatants from BMDCs stimulated in the absence of FBS were precipitated with 20 % (v/v) TCA and washed with acetone. Cell lysates were generated with RIPA buffer in the presence of a protease inhibitor cocktail. Cell lysates and precipitates from culture supernatants were electrophoresed, blotted and probed with anti-Caspase-1 (Adipogen, AG-20B-0042) or anti-IL-1β (Santa Cruz Biotechnol, sc-7884) antibodies.

#### THP-1 Transfection and Inflammasome Activation

THP-1 monocytes were differentiated into macrophages by treatment with 0.1 μM PMA for 48 hr. Macrophages (2.5 x 10^6^) were then detached using trypsine and nucleofected with the GFP or GFP-TMEM176B coding pcDNA1.3 plasmids using the Amaxa Cell Line Nucleofector Kit V-Lonza and nucleofector device (Amaxa). Sixteen hr later, cells were treated for 3 hr with 0.25 μg/ml LPS. Cells were washed and treated for 2 hr with 2.5 μM nigericin.

### Method Details

#### Cytosolic Ca^++^ Determination

BMDCs cultured on glass coverslips were loaded with 1 μM Fura-2 (ratiometric Ca^++^-sensitive probe) for 45 min in the dark. Cells were then washed and analyzed by time-lapse microscopy at 37°C. Fluorescence emission intensity at 510 nm was determined in individual wells alternating excitation wavelengths of 340 and 380 nm every 3 s. ATP was added when indicated at 0.5 mM.

#### *In Vivo* Cytotoxicity Assay

Splenocytes from naive C57BL/6 mice were stained alternatively with 0.8 (high) or 0.08 μM (low) DDAO-SE probe. The high DDAO population was loaded for 60 min at 37°C with 50 μM SIINFEKL OVA peptide. After three washes, the high and low population were mixed at 1:1 ratio. The mixed cells (2 x 10^6^) were injected i.v in WT, *Tmem176b*^*-/-*^ or *Tmem176b*^*-/-*^*Casp1*^*-/-*^ naive or tumor-bearing mice. Four hr later, mice were sacrificed and the spleens harvested. Splenocytes were analyzed by flow cytometry to assess DDAO high and low populations. Specific cytotoxicity was calculated using the following formula:

% specific lysis = (1-[r_naive_/r_tumor bearing_]) × 100

r = %DDAO^low^ cells / %DDAO^high^ cells

#### Screening of TMEM176B Inhibitors

CHO cells were transfected with pSecTag2B-PS-TMEM176A-mCherry and pSecTag2B-PS-TMEM176B-V5His plasmids using Lipofectamine 2000 for 4 hr, washed and cultured for 24 hr. Cells were then loaded with 1 μM ANG-2 for 30 min at 37°C, washed and incubated in 140 mM Na^+^-containing phosphate buffer or 140 mM NMDG to substitute Na^+^ in the presence of different doses of tested drugs or vehicle controls. Cells were then analyzed by flow cytometry using a BD Accuri C6 cytometer equipped with a 488 nm laser. ANG-2 emission was detected using a band-pass filter 530/30 and mCherry was determined using a 670 LP filter. FlowJo vX.0.7 software was used for data analysis. MFI from NMDG-containing solutions was subtracted to MFI from Na^+^-containing solutions. A maximum of two drugs was studied in each experiment. Screened drugs were from SCREEN-WELL® Ion Channel ligand library (Enzo Life Sciences; Farmingdale, NY).

#### Immunohistochemistry of Human Colon Microarrays

Expression of TMEM176B was analyzed by immunohistochemistry on 90 specimens of human colon tumors (US Biomax, Inc; Rockville, MD). Briefly, antigenic recovery was done by boiling slides in a pressure cooker for 10 min in the presence of alkaline buffer (10 mM Tris, 1 mM EDTA, 0.05% Tween 20, pH 9.0). Anti-TMEM176B antibody (2.5 μg/ml; Abcam, ab103929) or control rabbit IgG was incubated ON at 4°C. Staining was verified using EnVision+ System- HRP-labelled polymer anti-rabbit (Dako/Agilent, Santa Clara CA). Slides were counterstained with Meyer's hematoxylin, analyzed by two independent researchers in a blind fashion and categorized as high or low/negative TMEM176B expression in the stroma and parenchyma. Expression levels were then correlated with survival information provided by US Biomax.

#### Electrophysiology Experiments

Oocytes were surgically removed from MS222 (0.4%)-anesthetized *Xenopus laevis* female and dissociated under gentle agitation by a 2–3 hr incubation in an OR2 solution (82 mM NaCl; 2 mM KCl; 1 mM MgCl_2_; 5 mM HEPES pH 7.2) supplemented with collagenase 1A (1 mg/ml). Oocytes were then injected with 40 nl of *in vitro* synthesized *Tmem176b* mRNA at 1 μg/μl (mMessage mMachine Ultra kit). *Tmem176b* was fused to a signal peptide sequence (N-terminal) from pSecTag2B (Invitrogen, Carlsbad, CA) and to V5 + 6-His tags (C-terminal). The day after injection, oocytes were placed in a pH 8.0 solution (100 mM NaCl, 3 mM KCl, 2 mM MgCl_2_, 15 mM HEPES pH 8.0) changed daily. Two to three days later, currents were recorded in two-electrode voltage-clamp using a genclamp500 amplifier (Axon Inst., Foster City, CA) interfaced to a personal computer using the Digidata 1200 interface and the pClamp software (v 7.0; Axon Inst.). Prior to recording, oocytes were incubated in PMA at 0.1 μM in pH 8.0 solution for 20–30 min. Currents were filtered at 100 Hz and digitalized at 0.5 kHz before storage and further analysis. During recording, oocytes were continuously superfused with the pH 8.0 solution. The currents were quantified 5–15 min after holding the extracellular pH at 5.0. In TMEM176B-expressing oocytes, induction of an inward current was obtained by switching to a pH 5.0 solution.

#### Quantitative RT-PCR

Total RNA from tumors and lymph nodes was isolated using TRIzol Reagent (Invitrogen, Carlsbad, CA). Reverse transcription was performed using M-MLV Reverse Transcriptase and random primers following manufacturer’s instructions (Invitrogen). Gene expression was assessed with the Fast SYBR Green Master Mix reagent (Applied Biosystems, Foster City, CA). Mouse primers used in this study (Table S7) were all designed over different exons to prevent amplification of genomic DNA. Real-time PCR was performed using the ViiA 7 Real-Time PCR System (Applied Biosystems) or Eco Real-Time PCR System (Illumina). Gene expression was normalized using glyceraldehyde 3-phosphate dehydrogenase and expressed in arbitrary units using the 2−ΔΔCt method.

#### Gene Expression Analysis

Normalized NanoString nCounter data were analyzed from Chen et al. (2016). Gene expression data from Riaz et al (2017) were obtained from their GitHub repository (https://github.com/riazn/bms038_analysis/tree/master/data). RNA-seq count data were normalized to FPKM (fragment per kilobase per million) through the Bioconductor R package *DESeq2* 1.18.1. The on-treatment biopsy from patient 32 was excluded from further analyses since it presented extreme expression values.

#### CIBERSORT Analysis

The leukocyte signature matrix LM22 (547 genes) which discriminates 22 types of tumor-infiltrating immune cells was used for analysis. Normalized gene expression data from Riaz et al. (2017) cohort were processed with the CIBERSORT web tool (http://cibersort.stanford.edu/) setting no quantile normalization and 1.000 permutations as parameters. All samples were run with both relative and absolute modes. The first mode infers the relative cellular fraction for each cell of the LM22 matrix and the second calculates a score that reflects the absolute proportion of each cell type in the mixture.

#### Single Cell RNA-Seq Data Analysis

Normalized single cell expression data from Jerby-Arnon et al. (2018) was obtained from Gene Expression Omnibus (Accession number GSE115978). To study gene correlations, the expression matrix was processed with the software MAGIC (van Dijk et al., 2018) to deal with the undersampling of mRNA known as dropouts. R implementation of the MAGIC algorithm with default parameters (Rmagic v1.3.0) was applied. For correlation analysis, Spearman's Rank Correlation test was used.

#### Statistical Analyses

Statistical analyses were performed either by R project or GraphPad Prism 6 (GraphPad Software, Inc., La Jolla, CA). Survival analyses were done with the Log-rank (Mantel-Cox) test. Comparison of two experimental conditions was done with paired or unpaired Student’s *t* test. Comparison of multiple conditions was done with one or two-way ANOVA tests. Differences in gene expression and CIBERSORT scores between responder and non-responder groups were assessed using the unpaired *t*-test when normality assumption was met. Otherwise, Mann-Whitney *U* test was used. Differences between matched pre- and on-treatment samples were evaluated with paired *t*-test when normality assumption was met or otherwise with Wilcoxon signed-rank test. For correlation analysis, the Pearson coefficient was used when samples passed the normality test. Spearman coefficient was used for all other cases. Shapiro-Wilk test was performed to evaluate the normality assumption for all samples.

### Data and Software Availability

Mendeley dataset: https://data.mendeley.com/datasets/publish-confirmation/gvj6fc2b8v/1.

## Supplemental Information

Document S1. Figures S1–S8 and Tables S1–S7

Data S1. Screening of TMEM176B Inhibitors, Related to Figure 6

Document S2. Article plus Supplemental Information
