# Supplementary material for: Targeting TMEM176B Enhances Antitumor Immunity and Augments the Efficacy of Immune Checkpoint Blockers by Unleashing Inflammasome Activation
Source: Cancer Cell. 2019 May 13;35(5):767–781.e6. doi: 10.1016/j.ccell.2019.04.003 (PMC6521897; doi:10.1016/j.ccell.2019.04.003)
Supplement: Document S1. Figures S1–S8 and Tables S1–S7 [file mmc1.pdf]

Cancer Cell, Volume *35*

Supplemental Information

Targeting TMEM176B Enhances Antitumor Immunity and Augments the Efficacy of Immune Checkpoint Blockers by Unleashing Inflammasome Activation

Mercedes Segovia, Sofia Russo, Mathias Jeldres, Yamil D. Mahmoud, Valentina Perez, Maite Duhalde, Pierre Charnet, Matthieu Rousset, Sabina Victoria, Florencia Veigas, Cédric Louvet, Bernard Vanhove, R. Andrés Floto, Ignacio Anegon, Maria Cristina Cuturi, M. Romina Girotti, Gabriel A. Rabinovich, and Marcelo Hill

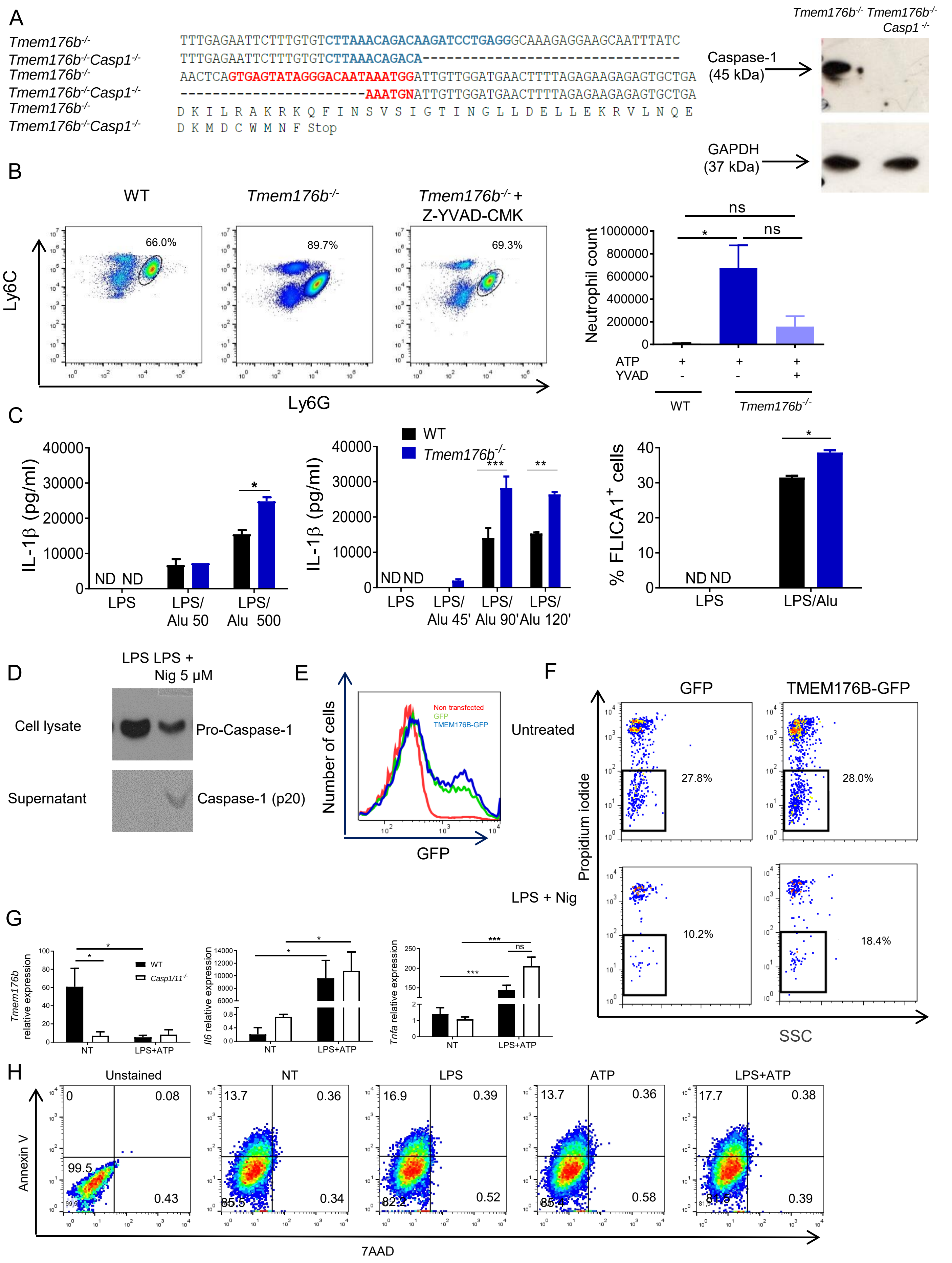
A
Tmem176b-/-
Tmem176b-/-Casp1-/-
TTTGAGAATTCTTTGTGTCTTAAACAGACAAGATCCTGAGGGCAAAGAGGAAGCAATTTATC
TTTGAGAATTCTTTGTGTCTTAAACAGACA------------------------------
AACTCAGTGAGTATAGGGACAATAAATGGATTGTTGGATGAACTTTTAGAGAAGAGAGTGCTGA
------------------------AAATGNATTGTTGGATGAACTTTTAGAGAAGAGAGTGCTGA
D K I L R A K R K Q F I N S V S I G T I N G L L D E L L E K R V L N Q E
D K M D C W M N F Stop
Tmem176b-/- Tmem176b-/- Casp1-/-
Caspase-1 (45 kDa)
GAPDH (37 kDa)
B
WT
Tmem176b-/-
Tmem176b-/- + Z-YVAD-CMK
66.0%
89.7%
69.3%
Ly6C
Ly6G
Neutrophil count
ATP
YVAD
WT
Tmem176b-/-
ns
C
IL-1β (pg/ml)
ND ND
LPS
LPS/ Alu 50
LPS/ Alu 500
WT
Tmem176b-/-
LPS/ Alu 45'
LPS/ Alu 90'
LPS/ Alu 120'
% FLICA1+ cells
LPS/Alu
D
LPS LPS + Nig 5 μM
Cell lysate
Supernatant
Pro-Caspase-1
Caspase-1 (p20)
E
Non transfected
GFP
TMEM176B-GFP
Number of cells
F
GFP
TMEM176B-GFP
Untreated
LPS + Nig
Propidium iodide
SSC
27.8%
28.0%
10.2%
18.4%
G
Tmem176b relative expression
Il6 relative expression
Tnfa relative expression
WT
Casp1/11-/-
NT
LPS+ATP
H
Unstained
NT
LPS
ATP
LPS+ATP
Annexin V
7AAD
0
0.08
99.5
0.43
13.7
0.36
85.5
0.34
16.9
0.39
82.2
0.52
13.7
0.36
85.4
0.58
17.7
0.38
81.5
0.39

Figure S1. Related to Figure 1.

(A) Sequence of genomic DNA (*Casp1* gene) from *Tmem176b*$^{-/-}$ and *Tmem176b*$^{-/-}$*Casp1*$^{-/-}$ mice. *Tmem176b*$^{-/-}$*Casp1*$^{-/-}$ (double KO) mice were generated by deletion of the indicated bases in *Casp1* gene in *Tmem176b*$^{-/-}$ mice using the CRISPR/Cas9 strategy. Proteins sequences are shown in the lower part of the alignment. Right: Western blot confirming the absence of Caspase-1 in *Tmem176b*$^{-/-}$*Casp1*$^{-/-}$ splenocytes.

(B) Absolute number of neutrophils (CD11b^{+}Ly6G^{+}Ly6C^{int}) determined by flow cytometry in 6-8 weeks-old male WT and *Tmem176b*$^{-/-}$ mice injected i.p with 20 mg/kg ATP. Four hr after ATP injection, peritoneal lavage was performed. In the plots, CD11b^{+} cells were analyzed for Ly6C and Ly6G expression. When indicated, the Caspase-1 inhibitor Z-YVAD-CMK was injected i.p at 5 mg/kg at the time of ATP treatment. At least six animals were studied in each group in two independent experiments. * p<0.05. ns: non significant. One-way ANOVA test. Representative scatter dot plots (left) and quantification for the different groups (right) are shown.

(C) Determination of IL-1β and Caspase-1 activation in WT and *Tmem176b*$^{-/-}$ bone marrow-derived DCs (BMDCs) treated with LPS (0.25 µg/ml) for 4 hr, washed and treated with 500 µg/ml of aluminum particles for the indicated times (left). Dose-response experiments are shown in the central graph. Culture supernatants were harvested and IL-1β was determined by ELISA (left and central graphs). Right: Caspase-1 activation was studied by flow cytometry using the FLICA1 reagent. BMDCs were stimulated for 3 hr with LPS and then incubated in the presence or absence of 500 µg/ml aluminum particles during 45 min. ND: not detected. * p<0.05; ** p<0.01 One-way ANOVA test. One experiment representative of three is shown.

(D) Western blot of Pro-Caspase-1 and Caspase-1 (p20) expression in cell lysates and culture supernatants from WT BMDCs primed for 3 hr with LPS (0.25 µg/ml), washed and treated or not with 5 µM nigericin (Nig) for 45 min. One experiment representative of two is shown.

(E) Transfection efficiency assessed by flow cytometry. THP-1 cells were differentiated to macrophages by treatment for 48 hr with 0.1 µM PMA. Cells were then electroporated with GFP or GFP-TMEM176B coding pcDNA1./8203 plasmids. Sixteen hr later, cells were left untreated or treated for 3 hr with 0.25 µg/ml LPS and then exposed for 2 hr to 2.5 µM nigericin (Nig).

(F) Cell viability assessed by flow cytometry of propidium iodide staining. One experiment representative of three is shown.

(G) Contribution of Caspase-1/11 to *Tmem176b, Tnfa* and *Il6* mRNA expression. WT and *Casp1/11*$^{-/-}$ BMDCs were left untreated (NT) or treated with LPS (0.25 µg/ml for 3 hr), washed and exposed to ATP (0.5 mM for 2 hr). *Tmem176b*, *Tnfa* and *Il6* mRNA expression was assessed by qRT-PCR. ns: non significant; * p<0.05; *** p<0,001. Two-way ANOVA test.

(H) Annexin V/7AAD staining of WT BMDCs either untreated (NT) or treated with LPS, ATP, or LPS plus ATP. The numbers indicate the percentage of cells in each quadrant. Data are representative of two independent experiments.

Mean ± SD are shown.

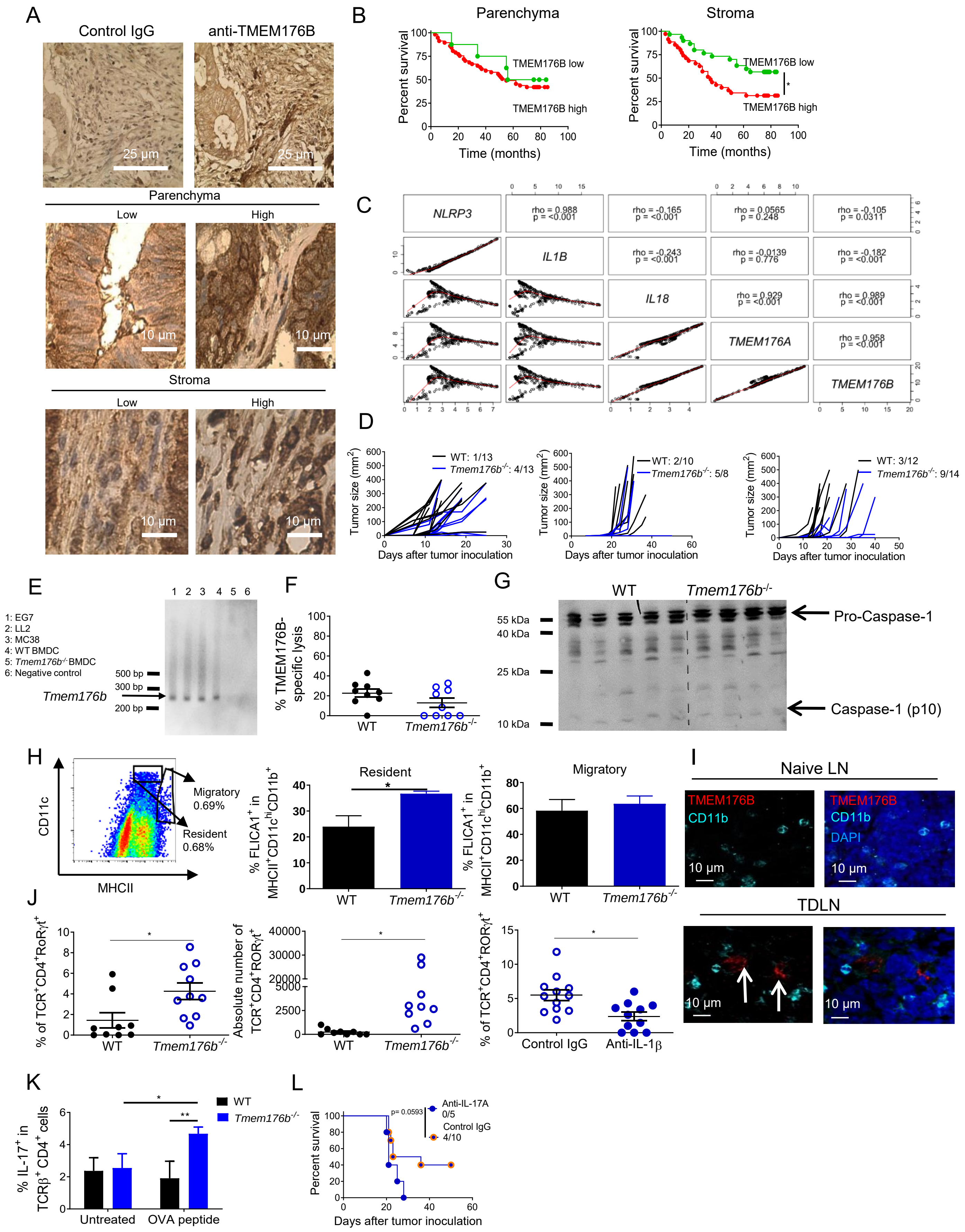

A
Control IgG
anti-TMEM176B
25 µm
Parenchyma
Low
High
10 µm
Stroma
B
Parenchyma
Stroma
Percent survival
TMEM176B low
TMEM176B high
Time (months)
*
C
NLRP3
IL1B
IL18
TMEM176A
TMEM176B
rho = 0.988 p = <0.001
rho = -0.165 p = <0.001
rho = 0.0565 p = 0.248
rho = -0.105 p = 0.0311
rho = -0.243 p = <0.001
rho = -0.0139 p = 0.776
rho = -0.182 p = <0.001
rho = 0.929 p = <0.001
rho = 0.989 p = <0.001
rho = 0.958 p = <0.001
D
Tumor size (mm²)
WT: 1/13
Tmem176b-/-: 4/13
WT: 2/10
Tmem176b-/-: 5/8
WT: 3/12
Tmem176b-/-: 9/14
Days after tumor inoculation
E
1: EG7
2: LL2
3: MC38
4: WT BMDC
5: Tmem176b-/- BMDC
6: Negative control
500 bp
300 bp
200 bp
Tmem176b
F
% TMEM176B-specific lysis
WT
Tmem176b-/-
G
WT
Tmem176b-/-
55 kDa
40 kDa
25 kDa
10 kDa
Pro-Caspase-1
Caspase-1 (p10)
H
CD11c
MHCII
Migratory 0.69%
Resident 0.68%
Resident
Migratory
% FLICA1+ in MHCII+CD11c^hiCD11b+
*
I
Naive LN
TMEM176B
CD11b
DAPI
10 µm
TDLN
J
% of TCR+CD4+RoRγt+
Absolute number of TCR+CD4+RORγt+
% of TCR+CD4+RORγt+
Control IgG
Anti-IL-1β
K
% IL-17+ in TCRβ+CD4+ cells
WT
Tmem176b-/-
Untreated
OVA peptide
**
L
Percent survival
p= 0.0593
Anti-IL-17A 0/5
Control IgG 4/10
Days after tumor inoculation

Figure S2. Related to Figure 2.

(A) Immunohistochemical staining of TMEM176B expression (brown staining, counterstained with hematoxylin) in human colon carcinomas samples (n=90). Representative images for parenchyma and stroma depicting low and high expression are shown. Scale bars, 10 or 25 µm.

(B) Survival analysis of colon cancer patients with high or low TMEM176B expression. Association of stromal ($p=0.0194$; Log-rank, Mantel-Cox test) and parenchimal ($p=0.55$; Log-rank, Mantel-Cox test) TMEM176B expression with overall survival. The staining and analysis were done by two independent researchers in a blinded fashion, ignoring the survival data for each sample.

(C) Matrix of scatterplots showing correlations between *NLRP3, IL1B, IL18, TMEM176A* and *TMEM176B* gene expression in 420 macrophages from single cell RNA-Seq data from melanoma biopsies (Jerby-Arnon et al., 2018). Correlations were made using Spearman's correlation coefficient. Red lines indicate the local regression (LOESS) fit; p, p value; rho, Spearman's correlation coefficient.

(D) Growth curves of 1×10^6 MC38 colon cancer cells (left), 1×10^5 LL2 lung cancer cells (center) or 1×10^6 EG7 thymic lymphoma cells (right) s.c injected into WT and *Tmem176b*$^{-/-}$ mice. Tumor growth was monitored every three days and measured in its longer and shorter diameters. Mice were euthanized when one of the diameters reached 2 cm. The ratio in the inset shows the number of animals developing tumors over the number of injected animals.

(E) *Tmem176b* mRNA expression in tumor cells and BMDCS analyzed by RT-PCR. The 249-bp band corresponds to the expected size of the specific amplified fragment. One experiment representative of two is shown.

(F) *In vivo* Tmem176b-specific cell lysis assessed as described in the STAR METHODS section. WT naive splenocytes were loaded either with low or high doses of DDAO and injected i.v into tumor-bearing WT and *Tmem176b*$^{-/-}$ animals 14 days after tumor inoculation. Four hr after injection, spleen was harvested and the ratio of low and high DDAO populations was studied to assess the percentage of specific cytotoxicity. Not significant. Student's *t* test.

(G) Analysis of Caspase-1 activation by Western blot comparing tumor lysates from WT and *Tmem176b*$^{-/-}$ animals. One experiment representative of two is shown.

(H) Representative scatter dot plot (left) for MHCII and CD11c expression within TDLN to identify migratory and resident cDCs. The central and right graphs depict the percentage of FLICA1^{+} cells (expressing active Caspase-1) within CD11b^{+} resident and migratory cDCs respectively from WT and *Tmem176b*$^{-/-}$ animals. One experiment representative of two is shown. * $p<0.05$ Student's *t* test.

(I) Immunostaining of TMEM176B^{+} CD11b^{int} cells in lymph nodes from naive mice or tumor-bearing animals (TDLN; harvested 14 days after EG7 tumor cell injection). Lymph nodes were immunostained with anti-TMEM176B (red) and anti-CD11b (Cyan) antibodies. Nuclei were stained with DAPI (blue). The white arrows indicate TMEM176B^{+} CD11b^{int} cells. At least three animals were studied in each group. Scale bars, 10 µm.

(J) Flow cytometry of TCRβ^{+} CD4^{+} RORγt^{+} T cells in TDLN from EG7-bearing WT and *Tmem176b*$^{-/-}$ mice. Relative (left) and absolute (central graphic) number of cells are shown. The right graph shows relative cell number of *Tmem176b*$^{-/-}$ animals treated with control IgG or anti-IL-1β neutralizing antibody. * $p<0.05$ Student's *t* test.

(K) Flow cytometry of IL-17A$^+$ CD4$^+$ T cells in TDLN cells from EG7-bearing WT and *Tmem176b*$^{-/-}$ mice. Animals. were euthanized 14 days after tumor cell inoculation. TDLN cells were re-stimulated *in vitro* with 10 µM OVA peptide 323-339 (ISQAVHAAHAEINEAGR) and IL-17A$^+$ CD4$^+$ T cells were determined. One experiment representative of three is shown. * $p<0.05$; ** $p<0.01$ Two-way ANOVA test.

(L) Survival of EG7-bearing *Tmem176b*$^{-/-}$ mice treated with control IgG or anti-IL-17A neutralizing antibody. $p=0.0593$. Log-rank (Mantel-Cox) test.

Mean ± SD are shown.

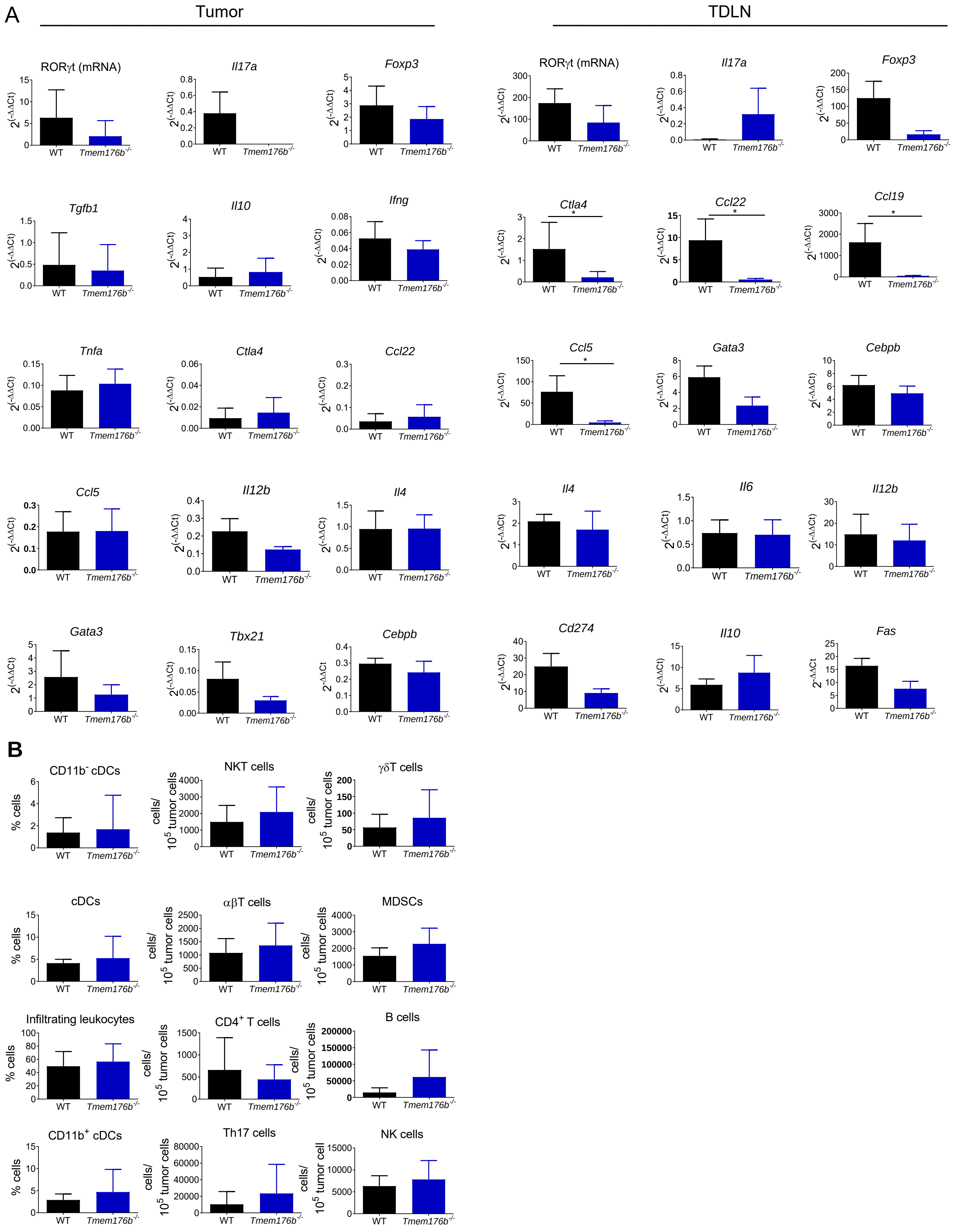
A
Tumor
TDLN
RORγt (mRNA)
Il17a
Foxp3
Tgfb1
Il10
Ifng
Tnfa
Ctla4
Ccl22
Ccl5
Il12b
Il4
Gata3
Tbx21
Cebpb
Ccl19
Il6
Cd274
Fas
2^(-ΔΔCt)
WT
Tmem176b-/-
*
B
CD11b- cDCs
NKT cells
γδT cells
cDCs
αβT cells
MDSCs
Infiltrating leukocytes
CD4+ T cells
B cells
CD11b+ cDCs
Th17 cells
NK cells
% cells
cells/ 10^5 tumor cells
cells/ 10^5 tumor cell

Figure S3. Related to Figure 2.

(A) Quantitative RT-PCR of the indicated transcripts in EG7 tumors and tumor-draining lymph nodes (TDLN) from WT and *Tmem176b*$^{-/-}$ mice harvested 14 days after tumor cell injection (n=5 per group). * p<0.05 Student's *t* test.

(B) Flow cytometry of the indicated markers (CD11b^{-} cDCs : TCRVβ12^{-} CD11c^{hi} MHC II^{+} CD11b^{-}; cDCs: TCRVβ12^{-} CD11c^{hi} MHC II^{+}; Infiltrating cells: TCRVβ12^{-} (EG7 cells are TCRVβ12+); CD11b^{+} cDCs: TCRVβ12^{-} CD11c^{hi} MHC II^{+} CD11b+; NKT cells: TCRVβ12^{-} TCRβ^{+} NK1.1^{+}; αβT cells: TCRVβ12^{-} TCRβ^{+}; CD4^{+} T cells: TCRVβ12^{-} TCRβ^{+} CD4^{+}; Th17: TCRVβ12^{-} TCRβ^{+}CD4^{+}RORγt^{+}; γδT cells :TCRVβ12^{-} TCRγδ^{+}; MDSCs: TCRβ^{-} CD11b^{+} Gr1^{+}; B cells: TCRVβ12-TCRβ^{-} CD19^{+}; NK cells: TCRVβ12^{-} TCRβ^{-} NK1.1^{+}. EG7 tumors from WT and *Tmem176b*$^{-/-}$ mice were harvested 14 days after tumor cell injection (at least n=5 per group). Tumors were disaggregated with collagenase D and cell suspensions were stained with the above mentioned antibodies. Data were analyzed by Student's *t* test.

Mean ± SD are shown.

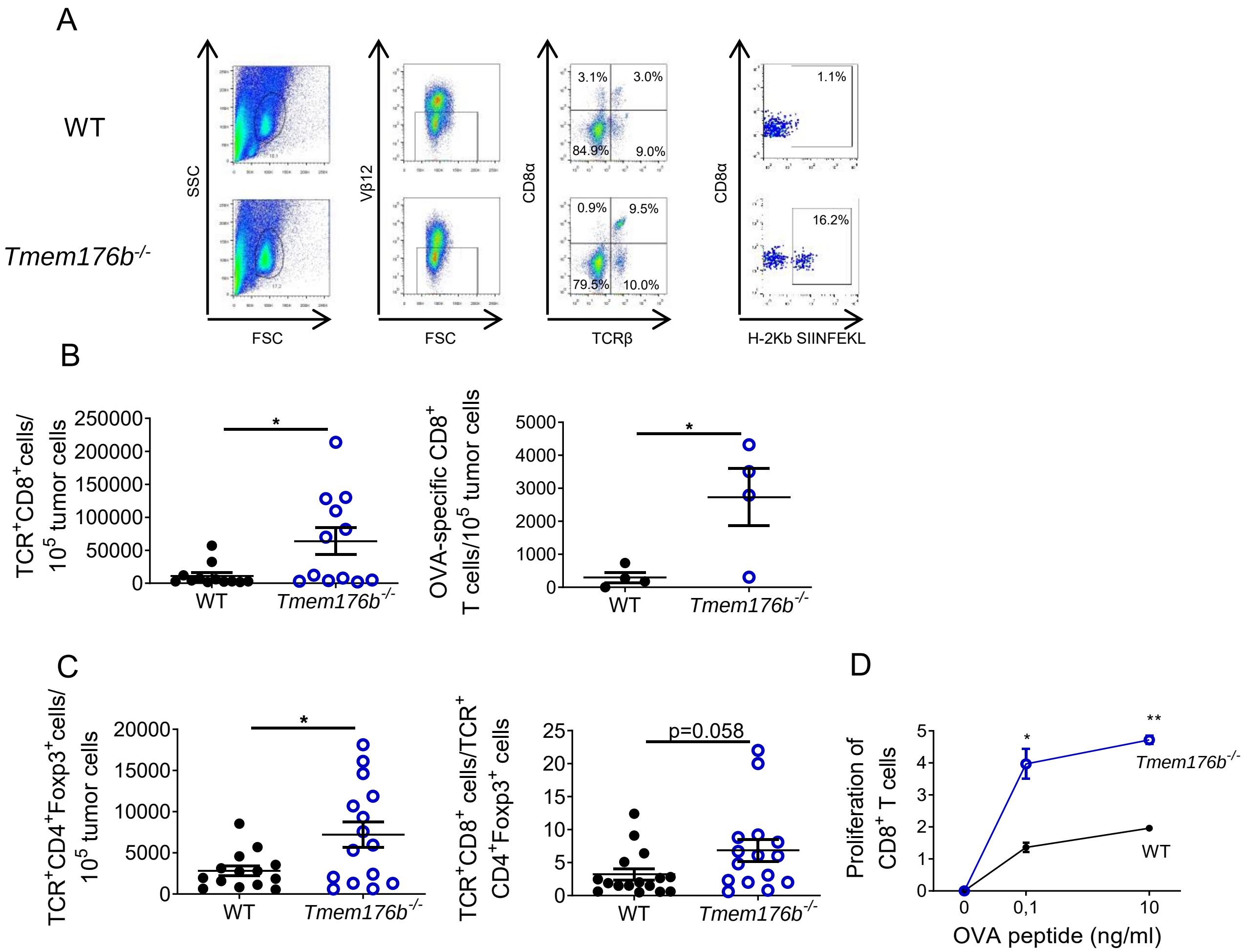

A
WT
Tmem176b-/-
SSC
FSC
Vβ12
FSC
CD8α
TCRβ
3.1%
3.0%
84.9%
9.0%
0.9%
9.5%
79.5%
10.0%
CD8α
H-2Kb SIINFEKL
1.1%
16.2%
B
TCR+CD8+cells/10^5 tumor cells
OVA-specific CD8+ T cells/10^5 tumor cells
*
WT
Tmem176b-/-
C
TCR+CD4+Foxp3+cells/10^5 tumor cells
TCR+CD8+ cells/TCR+CD4+Foxp3+ cells
p=0.058
D
Proliferation of CD8+ T cells
OVA peptide (ng/ml)
0
0,1
10
*
**
Tmem176b-/-
WT

Figure S4. Related to Figure 2.

(A) Flow cytometry analysis of total and OVA (SIINFEKL peptide)-specific $CD8^+$ T cells within the tumor microenvironment. TCRVβ12 staining was used to identify tumoral EG7 T cells. Representative of three experiments.

(B) Determination of the frequency of total and OVA-specific $CD8^+$ T cells in WT and *Tmem176b*$^{-/-}$ mice studied in A. * $p<0.05$ (Student's *t* test).

(C) Assessment of intratumoral regulatory T cells (Tregs) and CD8/Treg ratio within the tumor microenvironment. * $p<0.05$ (Student's *t* test).

(D) Proliferation of $CD8^+$ tumor-infiltrating T cells assessed by flow cytometry. Tumor-infiltrating T cells were purified by negative selection and re-stimulated *in vitro* in the presence of LPS-treated BMDCs (1/10 ratio) with SIINFEKL peptide. Proliferation of $CD8^+$ T cells was determined by flow cytometry by analyzing DDAO dilution. Four WT and four *Tmem176b*$^{-/-}$ animals were studied. * $p<0.05$; ** $p<0.01$. Student's *t* test.

Mean ± SD are shown.

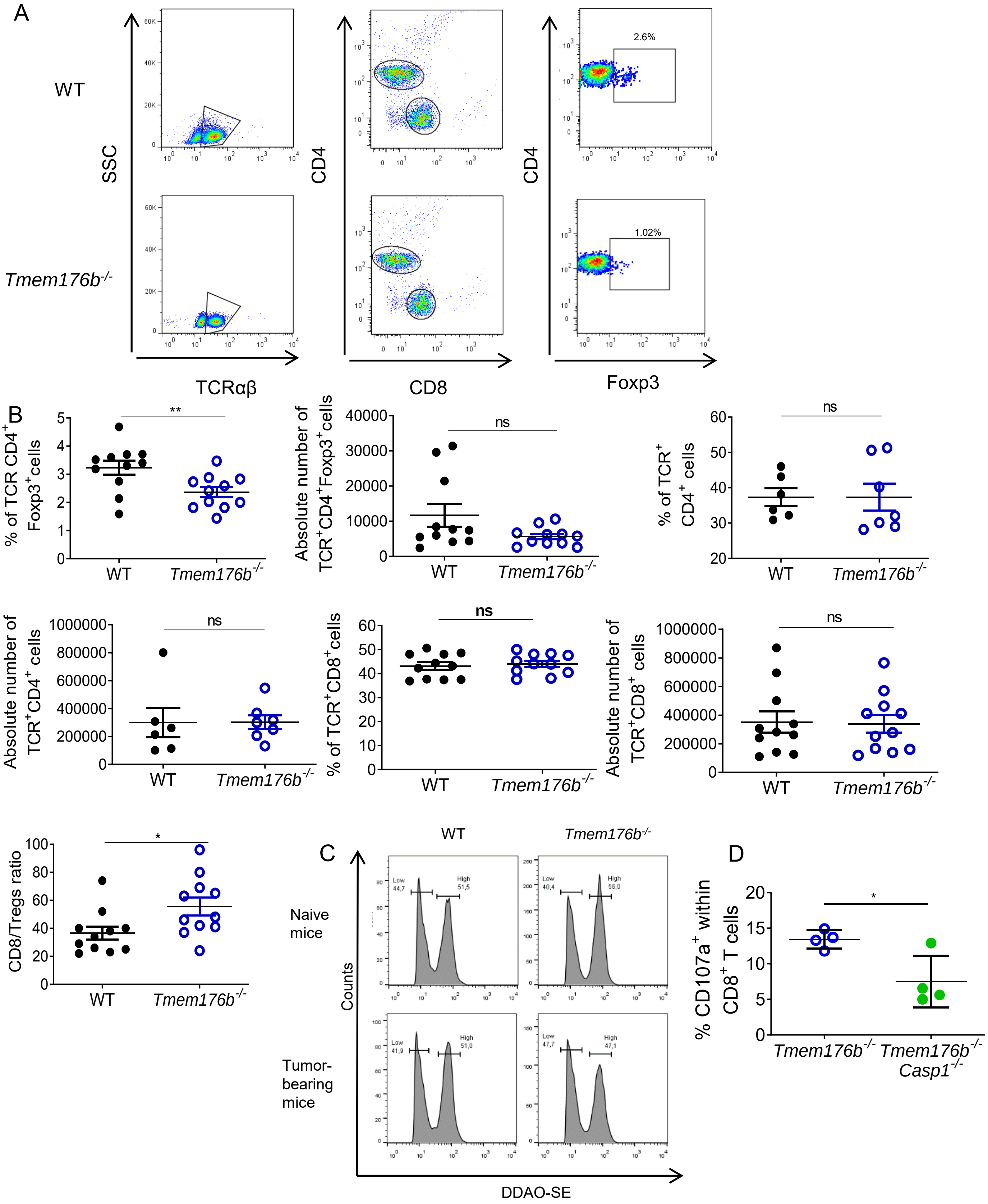
A
WT
Tmem176b-/-
SSC
TCRαβ
CD4
CD8
Foxp3
2.6%
1.02%
B
% of TCR CD4+ Foxp3+ cells
**
Absolute number of TCR+CD4+Foxp3+cells
ns
% of TCR+ CD4+ cells
Absolute number of TCR+CD4+ cells
% of TCR+CD8+cells
Absolute number of TCR+CD8+ cells
CD8/Tregs ratio
*
C
WT
Tmem176b-/-
Naive mice
Tumor-bearing mice
Counts
DDAO-SE
Low 44,7
High 51,5
Low 40,4
High 56,0
Low 41,9
High 51,0
Low 47,7
High 47,1
D
% CD107a+ within CD8+ T cells
Tmem176b-/-
Tmem176b-/- Casp1-/-

Figure S5. Related to Figure 2.

Tumor-draining lymph nodes from EG7-bearing WT and *Tmem176b*$^{-/-}$ animals were harvested 14 days after tumor inoculation. Different lymphocyte populations were analyzed by flow cytometry.

(A) Representative scatter dot plots indicating the frequency of cells expressing TCR$\alpha\beta$, CD4, CD8 and Foxp3.

(B) Percentage and absolute number of different lymphocyte populations. Student's *t* test. * $p<0.05$.

(C) Representative histograms of *in vivo* T-cell cytotoxicity against OVA-expressing cells shown in Figure 2G.

(D) Percentage of CD107a (degranulation marker) studied by flow cytometry within CD8$^+$ T cells infiltrating tumors in *Tmem176b*$^{-/-}$ and *Tmem176b*$^{-/-}$*Casp1*$^{-/-}$ mice. * $p<0.05$. Student's *t* test.

Mean ± SD are shown.

Table S1. Related to Figure 4. Analysis of data from Riaz *et al*. 2017. Paired analysis of inflammasome-associated gene expression profile in non-responders on/pre-treatment (anti-PD-1 antibody). IPI naive patients

Gene	p_value	fdr	fc[a]	p_value_log2	fdr_log2
TMEM176B	0.0390625	0.46875	0.7567829	0.029506455	0.37796936
TMEM176A	0.0390625	0.46875	0.72357185	0.048297486	0.37796936
CASP4	0.06761715	0.46875	0.3790591	0.111900929	0.44760372
IL18R1	0.08848316	0.46875	-0.42848366	0.175189823	0.54545455
NLRP6	0.09765625	0.46875	0.64605376	0.09765625	0.44760372
IL1RN	0.12890625	0.4921875	0.66854228	0.062994893	0.37796936
IL1RAP	0.1640625	0.4921875	0.3397688	0.25	0.54545455
IL1R2	0.1640625	0.4921875	0.62915261	0.053073014	0.37796936
IL1B	0.203125	0.54166667	0.39703187	0.31477899	0.62955798
CASP5	0.25	0.54545455	0.32829776	0.220629385	0.54545455
NLRP12	0.25	0.54545455	-0.78174959	0.25	0.54545455
AIM2	0.359375	0.71875	0.3707348	0.214068788	0.54545455
PYCARD	0.43022486	0.79426128	-0.17641233	0.477043954	0.74553571
GSDMD	0.49609375	0.85044643	0.07568183	0.588683156	0.74553571
IL18RAP	0.58736276	0.86979167	0.32970506	0.577301487	0.74553571
SIRT3	0.65103296	0.86979167	-0.06294952	0.607275156	0.74553571
IL1A	0.65234375	0.86979167	-0.01555178	0.65234375	0.74553571
IL18	0.65234375	0.86979167	0.14703032	0.55410695	0.74553571
ABHD5	0.8916341	1	0.01940535	0.786760725	0.85828443
CASP1	0.91015625	1	0.20761326	0.604608002	0.74553571
IL1R1	0.93720565	1	-0.02012097	0.65234375	0.74553571
NLRP7	0.94418251	1	0.16525862	0.833634883	0.86987988
NLRC4	0.95868982	1	0.01288561	0.645871386	0.74553571
NLRP3	1	1	-0.12646354	0.901745055	0.90174505

a: fc=FC=Log2(on-treatment/pre-treatment)

Table S2. Related to Figure 4. Analysis of data from Riaz *et al*. 2017. Inflammasome-related gene expression profile at pre-treatment stage (anti-PD-1). Total patients cohort.

Gene	p_value	fdr	fc[a]	p_value_log2	fdr_log2
IL18	0.26886624	0.9610583	-0.06039552	0.44313385	0.90452546
AIM2	0.30215351	0.9610583	-1.18605245	0.302153513	0.90452546
ABHD5	0.30818719	0.9610583	0.16813754	0.382450457	0.90452546
NLRP7	0.32975435	0.9610583	-0.65461498	0.329754349	0.90452546
NLRP6	0.36689235	0.9610583	-0.39904646	0.366892345	0.90452546
TMEM176A	0.43219326	0.9610583	-0.25246754	0.511560587	0.90452546
TMEM176B	0.50555314	0.9610583	-0.19952116	0.574971819	0.90452546
IL1RAP	0.51837548	0.9610583	-0.30412707	0.403383302	0.90452546
IL1R2	0.54450591	0.9610583	0.38787846	0.544505907	0.90452546
NLRP3	0.57126261	0.9610583	-0.195523	0.571262605	0.90452546
CASP1	0.5848672	0.9610583	0.1042929	0.571883087	0.90452546
IL1A	0.60894254	0.9610583	0.87696368	0.608942545	0.90452546
IL1B	0.62654098	0.9610583	0.51189721	0.626540976	0.90452546
IL1R1	0.62654098	0.9610583	0.50606896	0.600290705	0.90452546
IL18R1	0.63778549	0.9610583	-0.45389818	0.437045786	0.90452546
IL1RN	0.64070554	0.9610583	1.45651703	0.640705535	0.90452546
NLRP12	0.77138887	0.99608145	0.65653567	0.771388868	0.91889441
SIRT3	0.78870657	0.99608145	0.08908715	0.530108715	0.90452546
CASP5	0.80403261	0.99608145	-0.1828214	0.804032606	0.91889441
NLRC4	0.89734932	0.99608145	0.24808789	0.72127875	0.91889441
IL18RAP	0.91224132	0.99608145	0.18853179	0.912241316	0.9592106
GSDMD	0.91307466	0.99608145	-0.0650615	0.775336521	0.91889441
PYCARD	0.97625031	1	0.43830075	0.919243494	0.9592106
CASP4	1	1	0.04520392	1	1

a: fc=Log2(non responders/responders)

Table S3. Related to Figure 4. Analysis of data from Riaz *et al*. 2017. Inflammasome gene expression at pre-treatment stage (anti-PD-1). IPI naive patients.

Gene	p_value	fdr	fc[a]	p_value_log2	fdr_log2
IL1RAP	0.04919459	1	-0.84507093	0.068028757	0.97593334
CASP1	0.14290646	1	0.8346638	0.215221623	0.97593334
IL1R2	0.1895867	1	-0.27886838	0.189586695	0.97593334
ABHD5	0.2409666	1	0.28218828	0.336421084	0.97593334
NLRC4	0.25253619	1	0.57558152	0.427551872	0.97593334
PYCARD	0.28754702	1	0.786833	0.278688796	0.97593334
IL18R1	0.44913681	1	0.06558208	0.687783063	0.97593334
NLRP6	0.51709317	1	-0.31920005	0.517093172	0.97593334
IL1A	0.5180268	1	-1.37787825	0.518026796	0.97593334
AIM2	0.52539868	1	-1.62832169	0.285701311	0.97593334
IL1R1	0.66298319	1	0.20863927	0.525398683	0.97593334
NLRP3	0.69470252	1	0.02702505	0.694702525	0.97593334
IL1B	0.69470252	1	-1.04185706	0.694702525	0.97593334
GSDMD	0.69470252	1	-0.01225233	0.871411967	0.97593334
TMEM176A	0.73989814	1	-0.25420257	0.739898142	0.97593334
IL18RAP	0.73989814	1	0.22563589	0.739898142	0.97593334
NLRP12	0.73989814	1	1.41454798	0.739898142	0.97593334
CASP5	0.78594874	1	-0.32849091	0.78594874	0.97593334
NLRP7	0.92607981	1	-0.18311634	0.926079813	0.97593334
CASP4	0.96607684	1	-0.01321584	0.871781457	0.97593334
IL1RN	0.97593334	1	0.45616083	0.956178957	0.97593334
IL18	0.97593334	1	0.46255714	0.975933341	0.97593334
SIRT3	1	1	0.12306673	0.586578059	0.97593334
TMEM176B	1	1	-0.20950591	0.749503358	0.97593334

a: fc=Log2(non responders/responders)

Table S4. Related to Figure 4. Analysis of data from Riaz *et al.* 2017. Inflammasome gene expression at pre-treatment stage (anti-PD-1). IPI progressors patients.

Gene	p_value	fdr	fc[a]	p_value_log2	fdr_log2
NLRP7	0.11883877	0.87847857	-1.43340439	0.118838768	0.93807971
IL18	0.1981639	0.87847857	-0.41133656	0.237145531	0.93807971
IL18R1	0.27567576	0.87847857	-0.80848563	0.193730961	0.93807971
PYCARD	0.30505426	0.87847857	0.20522215	0.324660564	0.93807971
TMEM176A	0.44302914	0.87847857	-0.0569624	0.69003012	0.93807971
AIM2	0.49979366	0.87847857	-0.75123062	0.429215461	0.93807971
NLRP12	0.53313639	0.87847857	-0.11519728	0.533136387	0.93807971
TMEM176B	0.53995284	0.87847857	-0.01448501	0.756637021	0.93807971
IL1RN	0.53995284	0.87847857	2.1700564	0.388204956	0.93807971
NLRP3	0.60978261	0.87847857	-0.41559361	0.609782609	0.93807971
NLRP6	0.62191817	0.87847857	-0.37938013	0.621918166	0.93807971
SIRT3	0.66060345	0.87847857	0.09644427	0.60716929	0.93807971
IL18RAP	0.6777879	0.87847857	0.20423474	0.9658985	0.9658985
IL1B	0.68321676	0.87847857	2.29490109	0.68321676	0.93807971
NLRC4	0.72110363	0.87847857	-0.02883275	0.826361869	0.93807971
CASP1	0.72110363	0.87847857	-0.17541481	0.89899306	0.93807971
CASP4	0.72110363	0.87847857	0.15641347	0.721103627	0.93807971
IL1A	0.7546836	0.87847857	2.81678569	0.7546836	0.93807971
ABHD5	0.75627594	0.87847857	0.07287696	0.756880106	0.93807971
IL1R2	0.7988051	0.87847857	0.84095489	0.798805099	0.93807971
GSDMD	0.83902191	0.87847857	-0.05960373	0.71436234	0.93807971
IL1R1	0.87847857	0.87847857	0.77439692	0.828883091	0.93807971
IL1RAP	0.87847857	0.87847857	0.17601368	0.878478572	0.93807971
CASP5	0.87847857	0.87847857	-0.04991744	0.878478572	0.93807971

a: fc=Log2(non responders/responders)

Table S5. Related to Figure 4. Analysis of data from Riaz *et al*. 2017. Inflammasome-related gene expression at on-treatment stage (anti-PD-1). IPI naive patients.

Gene	p_value	fdr	fc[a]	p_value_log2	fdr_log2
TMEM176B	0,00390625	0,0625	1,84751874	0,00390625	0,0625
GSDMD	0,00541809	0,0625	0,63057312	0,00541809	0,0625
TMEM176A	0,0078125	0,0625	1,7844506	0,0078125	0,0625
NLRP6	0,01824504	0,07943254	0,80397817	0,01824504	0,07943254
IL18R1	0,01890336	0,07943254	0,88988129	0,01890336	0,07943254
IL1RAP	0,01985814	0,07943254	-0,890935	0,01985814	0,07943254
IL18RAP	0,02734375	0,09375	1,08256634	0,02734375	0,09375
CASP1	0,0546875	0,16193182	0,93111062	0,0546875	0,16193182
IL18	0,07344048	0,16193182	1,17698945	0,07344048	0,16193182
NLRP7	0,07421875	0,16193182	0,98046218	0,07421875	0,16193182
IL1R1	0,07421875	0,16193182	0,90803425	0,07421875	0,16193182
CASP4	0,0846027	0,1692054	0,60492518	0,0846027	0,1692054
PYCARD	0,15770119	0,29114067	0,60839068	0,15770119	0,29114067
NLRC4	0,25	0,42160536	0,91383868	0,25	0,42160536
NLRP3	0,26670139	0,42160536	0,55527122	0,26670139	0,42160536
CASP5	0,28107024	0,42160536	0,6440894	0,28107024	0,42160536
AIM2	0,30078125	0,42463235	0,22745745	0,30078125	0,42463235
IL1A	0,359375	0,47916667	0,67605275	0,359375	0,47916667
IL1R2	0,42578125	0,53782895	0,82922261	0,42578125	0,53782895
NLRP12	0,5226743	0,62720916	0,47842529	0,5226743	0,62720916
ABHD5	0,74473826	0,85112944	-0,0715091	0,74473826	0,85112944
IL1RN	0,8203125	0,89488636	0,98637591	0,8203125	0,89488636
SIRT3	0,97073348	1	0,00631734	0,97073348	1
IL1B	1	1	0,92505986	1	1

a: fc=Log2(non responders/responders)

Table S6. Related to Figure 4. Analysis of data from Riaz *et al*. 2017. Paired analysis of Inflammasome-related gene expression in responders (anti-PD-1) on/pre-treatment stage. IPI naive patients.

Gene	p_value	fdr	fc	p_value_log2	fdr_log2
IL18R1	0,02143359	0,51440626	-0,4859683	0,021433594	0,51440626
IL1RAP	0,12932599	0,86453951	0,17706561	0,129325989	0,864539513
NLRP12	0,22875214	0,86453951	-0,066435	0,228752136	0,864539513
NLRP7	0,28588144	0,86453951	-0,2509041	0,285881445	0,864539513
IL1R2	0,30379486	0,86453951	0,16216661	0,303794861	0,864539513
GSDMD	0,30379486	0,86453951	0,00292277	0,303794861	0,864539513
ABHD5	0,36921692	0,86453951	0,21108858	0,369216919	0,864539513
CASP4	0,36921692	0,86453951	0,0423972	0,369216919	0,864539513
NLRP3	0,43614622	0,86453951	-0,1577218	0,43614622	0,864539513
IL1RN	0,44229889	0,86453951	0,32536957	0,442298889	0,864539513
IL18RAP	0,44229889	0,86453951	0,16380521	0,442298889	0,864539513
SIRT3	0,46035442	0,86453951	-0,0661062	0,460354416	0,864539513
IL1B	0,46829224	0,86453951	0,21193413	0,468292236	0,864539513
IL18	0,5508728	0,88139648	-0,0953584	0,550872803	0,881396484
TMEM176A	0,5508728	0,88139648	-0,422569	0,550872803	0,881396484
IL1R1	0,60945892	0,90792501	0,02509495	0,609458923	0,907925011
NLRC4	0,75676165	0,90792501	-0,0565318	0,756761647	0,907925011
PYCARD	0,76602936	0,90792501	-0,4921455	0,766029358	0,907925011
TMEM176B	0,79870605	0,90792501	-0,3214791	0,798706055	0,907925011
CASP1	0,79914976	0,90792501	0,03520439	0,799149764	0,907925011
AIM2	0,80196793	0,90792501	0,06147296	0,801967933	0,907925011
NLRP6	0,83226459	0,90792501	-0,080559	0,832264593	0,907925011
IL1A	0,88706869	0,9256369	-0,0472519	0,887068694	0,925636898
CASP5	1	1	-0,2518302	1	1

a: fc=FC=Log2(on-treatment/pre-treatment)

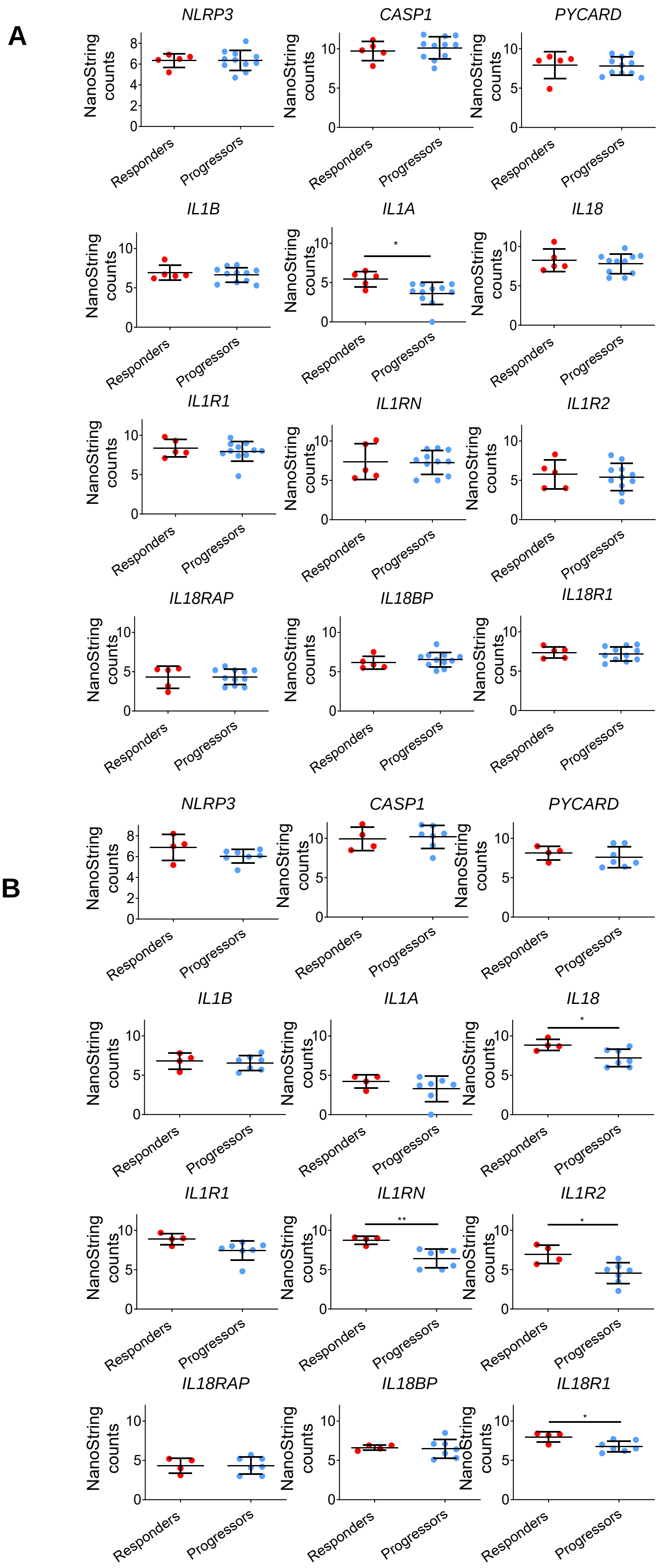
A
B
NLRP3
CASP1
PYCARD
IL1B
IL1A
IL18
IL1R1
IL1RN
IL1R2
IL18RAP
IL18BP
IL18R1
NanoString counts
Responders
Progressors

Figure S6. Related to Figure 5.

The log2-transformed normalized NanoString counts from melanoma tumor biopsies for the indicated inflammasome-related genes is shown (Chen *et al*. 2016 cohort analyzed in Figure 5). Biopsies were obtained before anti-CTLA-4 therapy in A and B.

(A) Patients were classified as responders and progressors to anti-CTLA-4 therapy according to clinical outcome as defined by Chen *et al* (2016). * $p<0.05$. Non-paired Student's *t* test.

(B) Patients progressing to anti-CTLA-4 therapy were then treated with anti-PD-1 antibodies. Based on their clinical outcome (with regards to anti-PD-1 therapy), they were classified as responders and progressors. * $p<0.05$; ** $p<0.01$. Non-paired Student's *t* test.

Mean ± SD are shown.

A

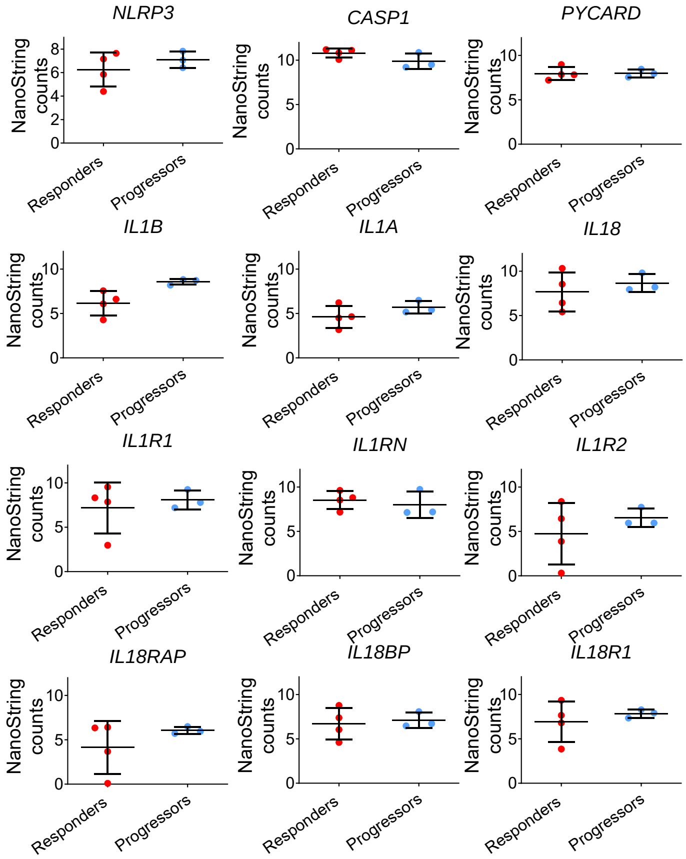
NLRP3
CASP1
PYCARD
IL1B
IL1A
IL18
IL1R1
IL1RN
IL1R2
IL18RAP
IL18BP
IL18R1
NanoString counts
Responders
Progressors

B

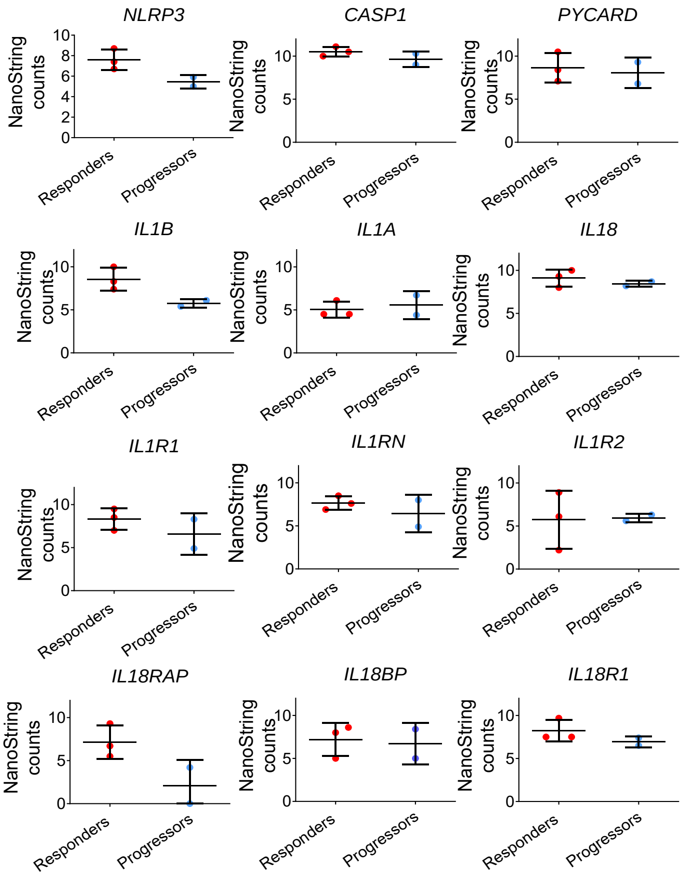
NLRP3
CASP1
PYCARD
IL1B
IL1A
IL18
IL1R1
IL1RN
IL1R2
IL18RAP
IL18BP
IL18R1
NanoString counts
Responders
Progressors

Figure S7. Related to Figure 5.

The log2-transformed normalized NanoString counts from melanoma tumor biopsies for the indicated inflammasome-related genes is shown for melanoma patients from the Chen *et al*. 2016 cohort analyzed (in Figure 5). Non-paired Student's *t* test.

(A) Tumor biopsies were obtained before anti-PD-1 therapy in patients not responding to anti-CTLA-4 antibodies. In the figure, responders and progressors were classified according to their clinical outcome in response to anti-PD-1 therapy.

(B) Tumor biopsies were obtained during the anti-CTLA-4 therapy (first 2-3 months). Responders and progressors were classified according to their clinical outcome in response to anti-CTLA-4 therapy.

Mean ± SD are shown.

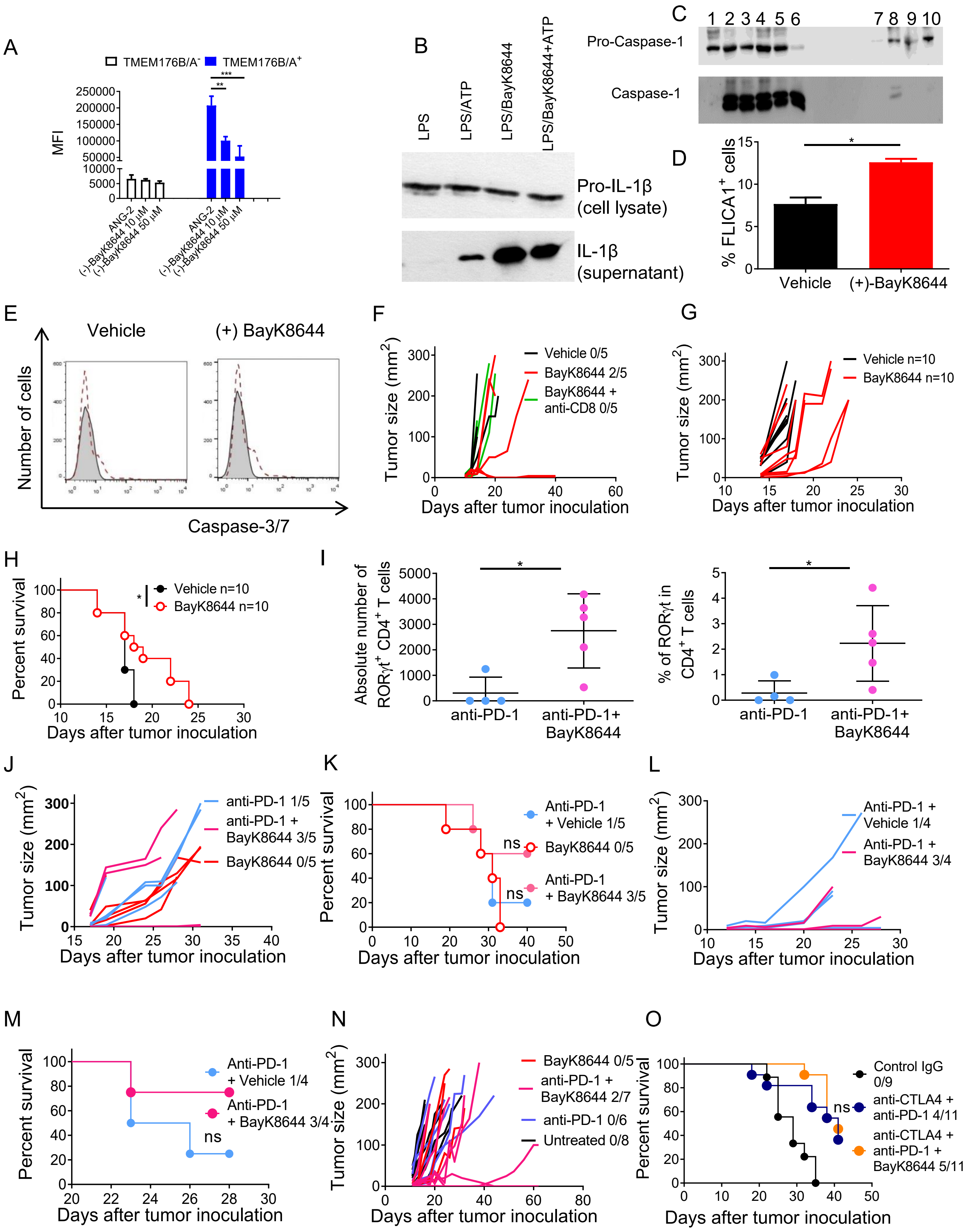
A
TMEM176B/A⁻ TMEM176B/A⁺
MFI
ANG-2
(-)-BayK8644 10 μM
(-)-BayK8644 50 μM
B
LPS
LPS/ATP
LPS/BayK8644
LPS/BayK8644+ATP
Pro-IL-1β (cell lysate)
IL-1β (supernatant)
C
Pro-Caspase-1
Caspase-1
D
% FLICA1⁺ cells
Vehicle
(+)-BayK8644
E
Vehicle
(+) BayK8644
Number of cells
Caspase-3/7
F
Vehicle 0/5
BayK8644 2/5
BayK8644 + anti-CD8 0/5
Tumor size (mm²)
Days after tumor inoculation
G
Vehicle n=10
BayK8644 n=10
H
Percent survival
Vehicle n=10
BayK8644 n=10
I
Absolute number of RORγt⁺ CD4⁺ T cells
% of RORγt in CD4⁺ T cells
anti-PD-1
anti-PD-1+ BayK8644
J
anti-PD-1 1/5
anti-PD-1 + BayK8644 3/5
BayK8644 0/5
K
Anti-PD-1 + Vehicle 1/5
BayK8644 0/5
Anti-PD-1 + BayK8644 3/5
ns
L
Anti-PD-1 + Vehicle 1/4
Anti-PD-1 + BayK8644 3/4
M
Anti-PD-1 + Vehicle 1/4
Anti-PD-1 + BayK8644 3/4
ns
N
BayK8644 0/5
anti-PD-1 + BayK8644 2/7
anti-PD-1 0/6
Untreated 0/8
O
Control IgG 0/9
anti-CTLA4 + anti-PD-1 4/11
anti-CTLA4 + anti-PD-1 + BayK8644 5/11
ns

Figure S8. Related to Figure 6.

(A) Analysis of BayK8644 activity on TMEM176B/A-transfected cells. CHO-7 cells were transfected with TMEM176B and TMEM176A-mcherry-coding pcDNA1.3 plasmids. Cells were then loaded with the Na^+-sensitive fluorescent dye Asante NaTRIUM Green 2 (ANG-2). The graph indicates quantification of ANG-2 mean fluorescence intensity (MFI) subtracting in each condition the MFI obtained in Na^+-free buffer. Untreated and (-) BayK8644-treated cells were studied. One experiment representative of three is shown. ** $p<0.01$; *** $p<0.001$. Two-way ANOVA test.

(B) Western blot of Pro-IL-1β and IL-1β expression. BMDCs were treated for 3 hr with 0.25 µg/ml LPS. Cells were washed and then treated with 2 mM ATP, 2.5 µM BayK8644 or both stimuli. Cell lysates and precipitated culture supernatants were electrophoresed, blotted and analyzed using an anti-IL-1β antibody. One experiment representative of four is shown.

(C) Western blot analysis of Pro-Caspase-1 and Caspase-1 expression in BMDCs (supernatants) treated as follows. 1: LPS; 2: LPS/ATP; 3: LPS/verapamil + ATP; 4: LPS/nifedipine + ATP; 5: LPS/diltiazem + ATP; 6: LPS/DMSO + ATP; 7: LPS/ATP medium standard K^+; 8: LPS/BayK8644 medium standard K^+; 9: LPS/ATP medium high K^+; 10: LPS/BayK medium high K^+. One experiment representative of two is shown.

(D) Flow cytometry of active Caspase-1 in BMDCs treated with 5 µM BayK8644 for 2 hr and then stained with FLICA1. Student's *t* test. * $p<0.05$. One experiment representative of three is shown.

(E) Number of apoptotic EG7 tumor cells treated *in vitro* with vehicle (ethanol) or with (+) BayK8644 (10 µM). Apoptosis was determined by analyzing active caspase-3/7. The grey histogram shows unstained conditions and the dotted line shows caspase-3/7 staining. One experiment representative of three is shown.

(F) Growth of EG7 tumor cells inoculated in WT mice treated or not with BayK8644 in the absence or presence of anti-CD8 depleting antibody. Growth of individual tumors is shown.

(G-H) Growth of individual tumors (G) and survival (H) of BALB/c mice injected s.c with $1x10^5$ CT26 colon cancer cells. Mice were treated daily i.p with vehicle or 1 mg/kg BayK8644 at days 3-15 after tumor cell inoculation. * $p<0.05$; Log-rank (Mantel-Cox) test.

(I) Absolute number (left) and percentage (right) of $TCR\beta^+CD4^+ROR\gamma t^+$ T cells within TDLN from tumor (EG7)-bearing mice treated with anti-PD-1 or anti-PD-1 + BayK8644. Anti-PD-1 antibody (250 µg) was injected i.p at days 6, 9 and 12 after tumor inoculation. BayK8644 was injected every day since day 9 (in mice with established tumors) until day 21. * $p<0.05$ Student's *t* test.

(J-K) Growth of individual tumors (J) and survival (K) of C57BL/6 mice injected s.c with 1×10^5 LL/2 lung tumor cells. WT mice were injected with LL/2 cells and then treated with 250 µg anti-PD-1 antibody at days 6, 9 and 12 after tumor inoculation. BayK8644 was injected daily since day 9 (tumors were 10-20 mm^2 in surface) until day 21. In this therapeutic protocol BayK8644 monotherapy showed no anti-tumor effect. ns: non significant. Log-rank (Mantel-Cox) test.

(L-M) Growth of individual tumors (L) and survival (M) of C57BL/6 mice injected s.c with 1×10^6 MC38 colon cancer cells. WT mice were injected with MC38 cells and then treated with 250 µg anti-PD-1 antibody at days 6, 9 and 12 after tumor inoculation. BayK8644 was injected daily since day 9 (tumors were 10-20 mm^2 in surface) until day 21. Log-rank (Mantel-Cox) test.

(N) Growth of 5555 melanoma cells in WT mice treated or not with anti-PD-1 antibody (days 6, 9 and 12), BayK8644 (days 9-21) or both. All animals had established tumors when BayK8644 treatment was started. Growth of individual tumors is shown.

(O) Survival of C57BL/6 mice inoculated s.c with 2.5 x 10^5 5555 melanoma cells and receiving combination treatments. Ten days after tumor cell inoculation, animals were treated with: a) control IgG; b) anti-CTLA-4 mAb + anti-PD-1 mAb or c) anti-CTLA-4 mAb + anti-PD-1 mAb + BayK8644. Mice were sacrificed when one of the tumor diameters reached 2 cm. Mice survival was monitored. Statistical significance was determined using the Log-rank (Mantel-Cox) test. ns: non significant. Control IgG vs anti-CTLA-4 + anti-PD-1 $p= 0.0057$; Control IgG vs anti-CTLA-4 + anti-PD-1 + BayK8644 $p<0.0001$; anti-CTLA-4 + anti-PD-1 vs anti-CTLA4 + anti-PD-1 + BayK8644, ns.

Mean ± SD are shown.

Table S7. Related to STAR METHODS. List of oligonucleotides.

Gene or mRNA	Primer forward	Primer reverse
RORγt (mRNA)	GGA GGA CAG GGA GCC AAG TT	AGT AGG CCA CAT TAC ACT GCT
Il17a	AGT CCA GGG AGA GCT TCA TCT	TCT TCA TTG CGG TGG AGA GTC
Foxp3	TCC AAG TCT CGT CTG AAG GC	GCG AAA GTG GCA GAG AGG TA
Tgfb1	TGA CGT CAC TGG AGT TGT ACG G	GGT TCA TGT CAT GGA TGG TGC
Il10	CCA AGC CTT ATC GGA AAT GA	TTT TCA CAG GGG AGA AAT CG
Ifng	TGG CTC TGC AGG ATT TTC ATG	TCA AGT GGC ATA GAT GTG GAA GAA
Tnfa	TGG GAG TAG ACA AGG TAC AAC CC	CAT CTT CTC AAA ATT CGA GTG ACA A
Ctla4	CTG AAG GTT GGG TCA CCT GT	TGG ACT CCG GAG GTA CAA AG
Ccl22	CAC CCT CTG CCA TCA CGT TT	CCT GGG ATC GGC ACA GAT AT
Ccl5	ACT CCC TGC TGC TTT GCC TAC	GAG GTT CCT TCG AGT GAC A
Il12b	GGA AGC ACG GCA GCA GAA TA	AAC TTG AGG GAG AAG TAG GAA TGG
Il4	GGT CTC AAC CCC CAG CTA GT	GCC GAT GAT CTC TCT CAA GTG AT
Gata3	AGG ATG TCC CTG CTC TCC TT	GCC TGC GGA CTC TAC CAT AA
Tbx21	GTC TGG GAA GCT GAG AGT CG	CTT TCC ACA CTG CAC CCA CT
Cebpb	GGA GAC GCA GCA CAA GGT	AGC TGC TTG AAC AAG TTC CG
Ccl19	GAC CTT CCC AGC CCC AAC T	CGG AAG GCT TTC ACG ATG TT
Il6	GAG GAT ACC ACT CCC AAC AGA CC	AAG TGC ATC ATC GTT GTT CAT ACA
Fas	AGT TTC ATG AAC CCG CCT C	GCA GAC ATG CTG TGG ATC TG
Cd274	ATG CTC AGA AGT GGC TGG AT	TGC TGC ATA ATC AGC TAC GG
Tmem176b	ACT CCA GCT AGA ATT GCC ACA G	CAT CAG CAT CCA CAT CCA CC
Gapdh	CTA CAG CAA CAG GGT GGT GG	TAT GGG GGT CTG GGA TGG
