# Supplementary material for: Targeting TMEM176B Enhances Antitumor Immunity and Augments the Efficacy of Immune Checkpoint Blockers by Unleashing Inflammasome Activation
Source: Cancer Cell. 2019 May 13;35(5):767–781.e6. doi: 10.1016/j.ccell.2019.04.003 (PMC6521897; doi:10.1016/j.ccell.2019.04.003)

## Slide 1
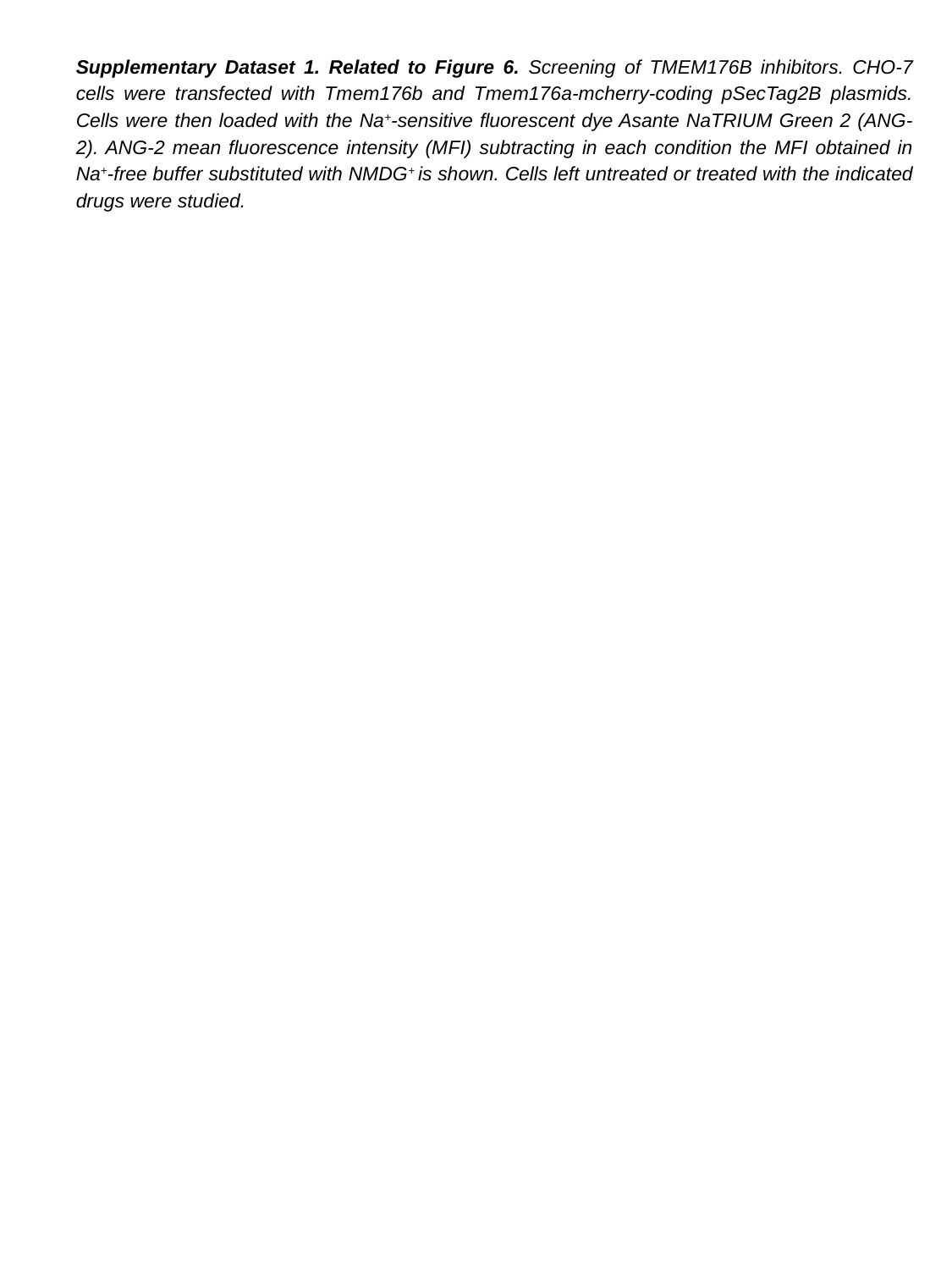

Supplementary Dataset 1. Related to Figure 6. Screening of TMEM176B inhibitors. CHO-7 cells were transfected with Tmem176b and Tmem176a-mcherry-coding pSecTag2B plasmids. Cells were then loaded with the Na+-sensitive fluorescent dye Asante NaTRIUM Green 2 (ANG-2). ANG-2 mean fluorescence intensity (MFI) subtracting in each condition the MFI obtained in Na+-free buffer substituted with NMDG+ is shown. Cells left untreated or treated with the indicated drugs were studied.

## Slide 2
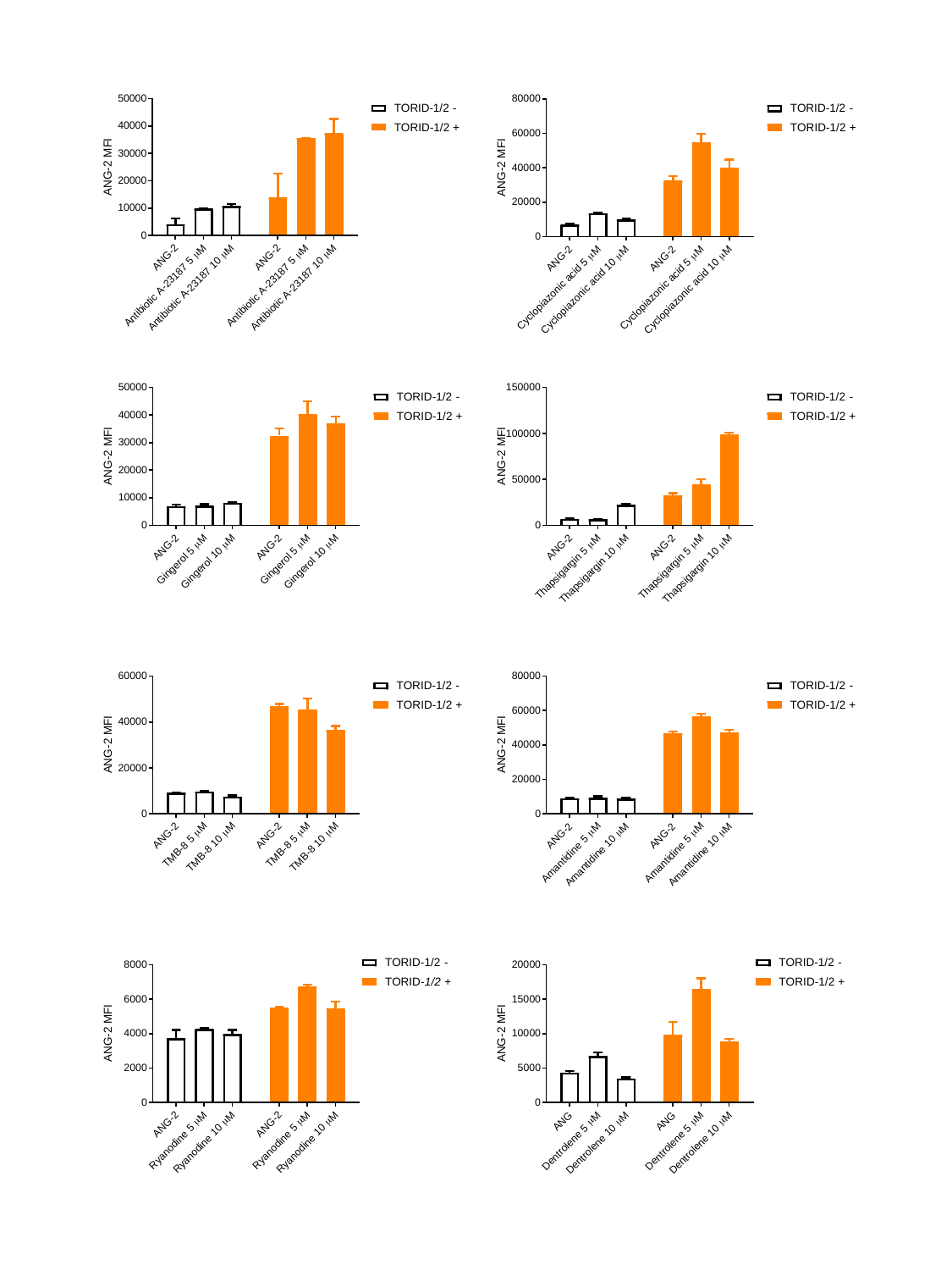

## Slide 3
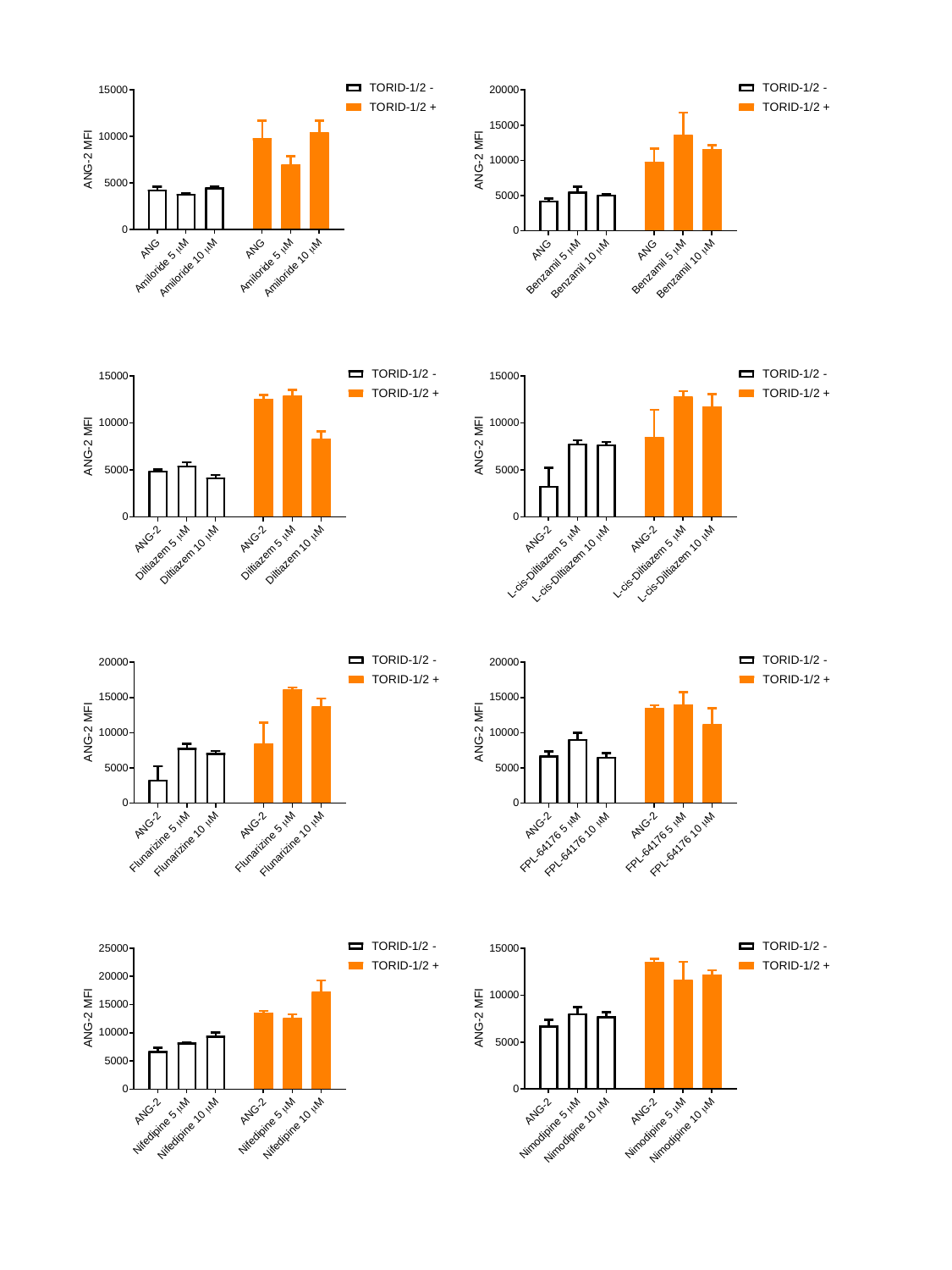

## Slide 4
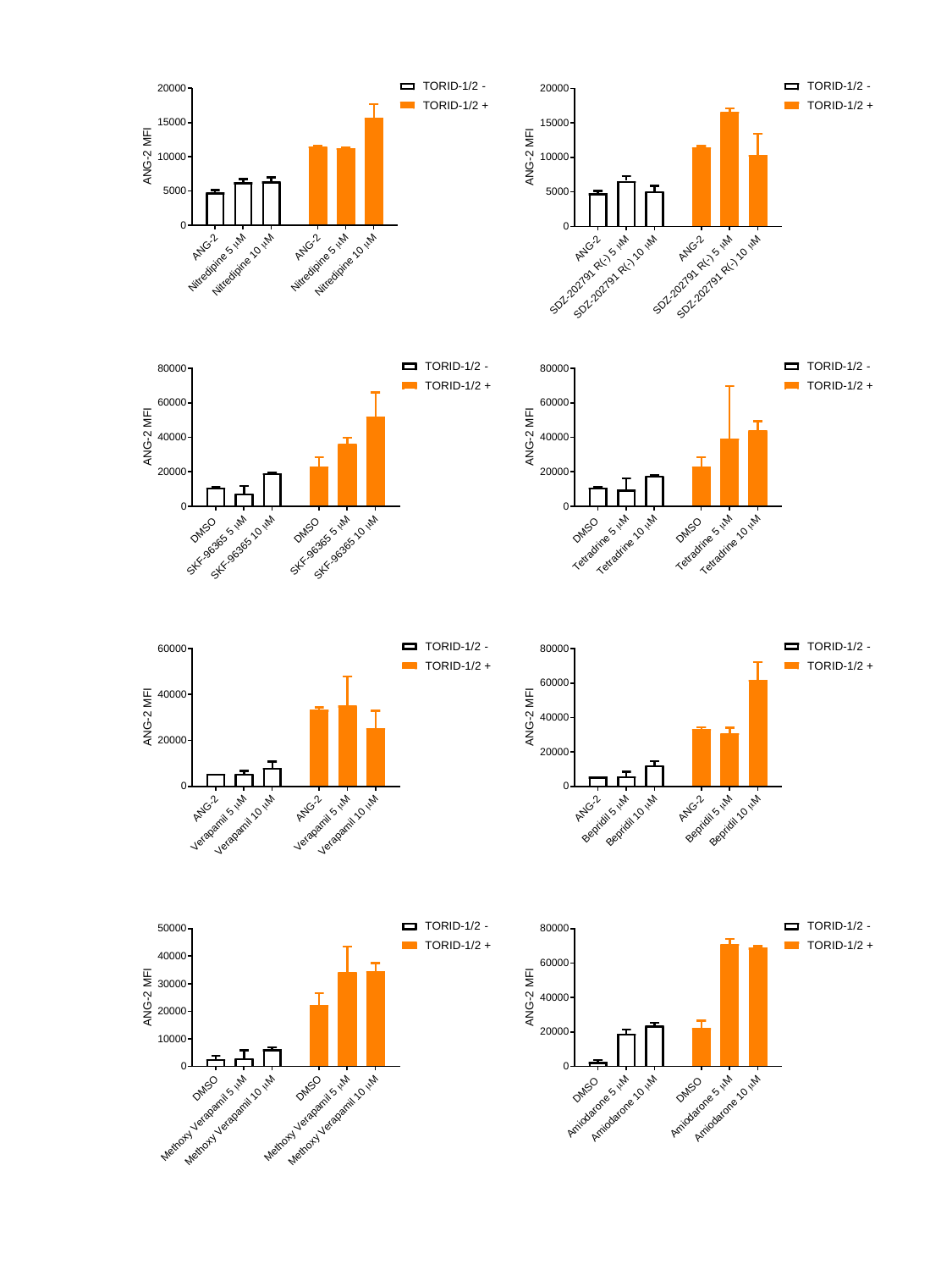

## Slide 5
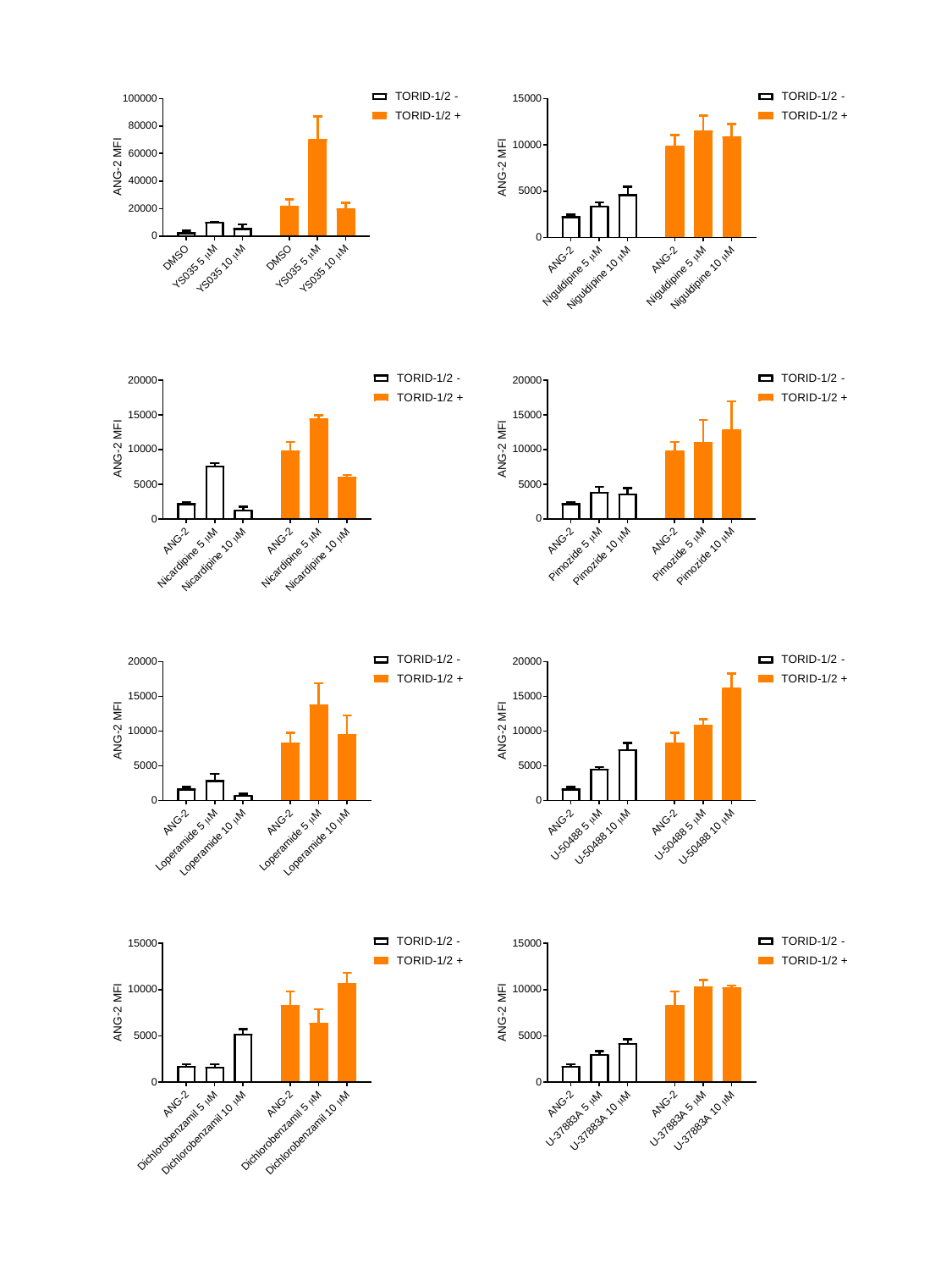

## Slide 6
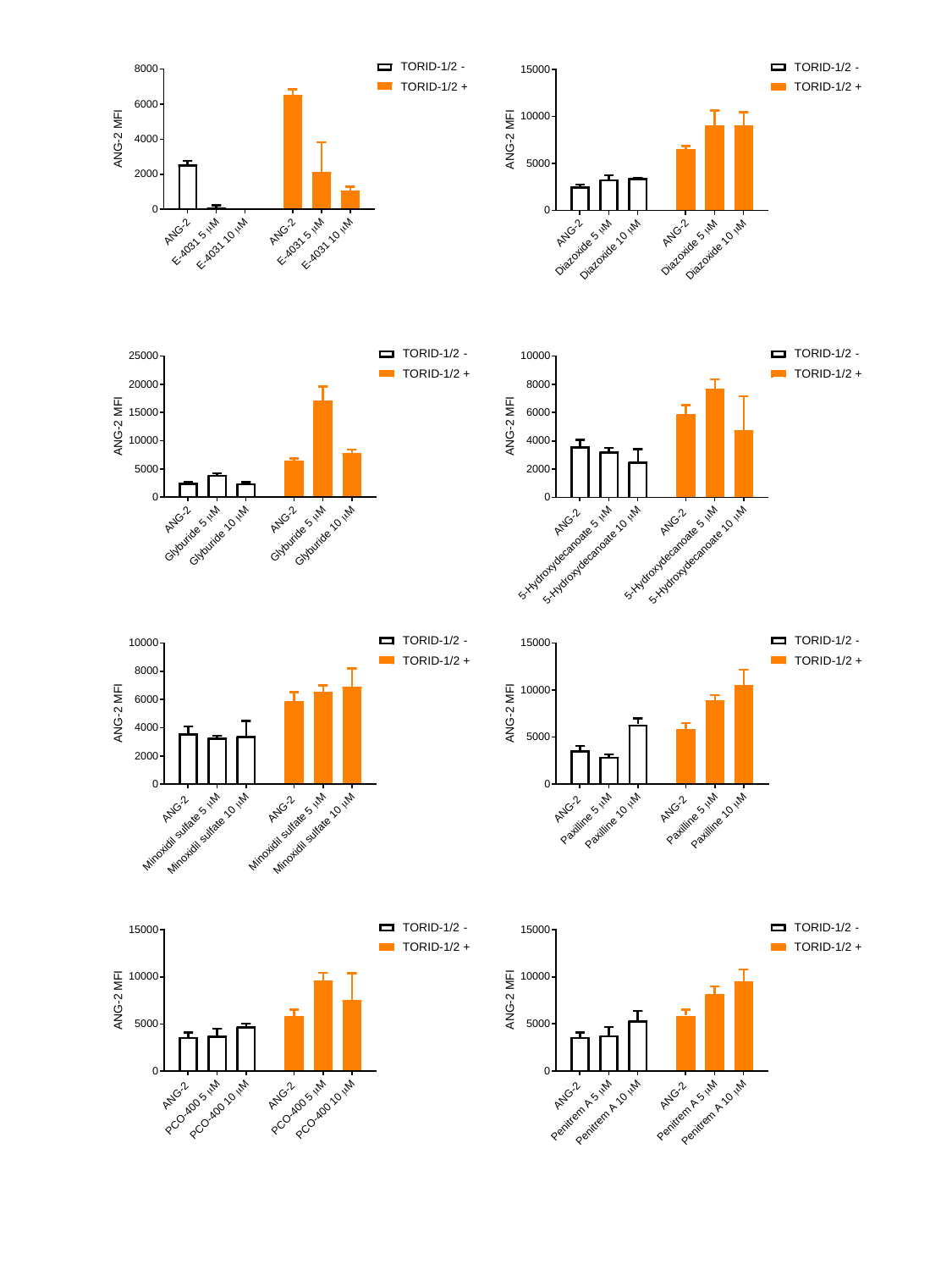

## Slide 7
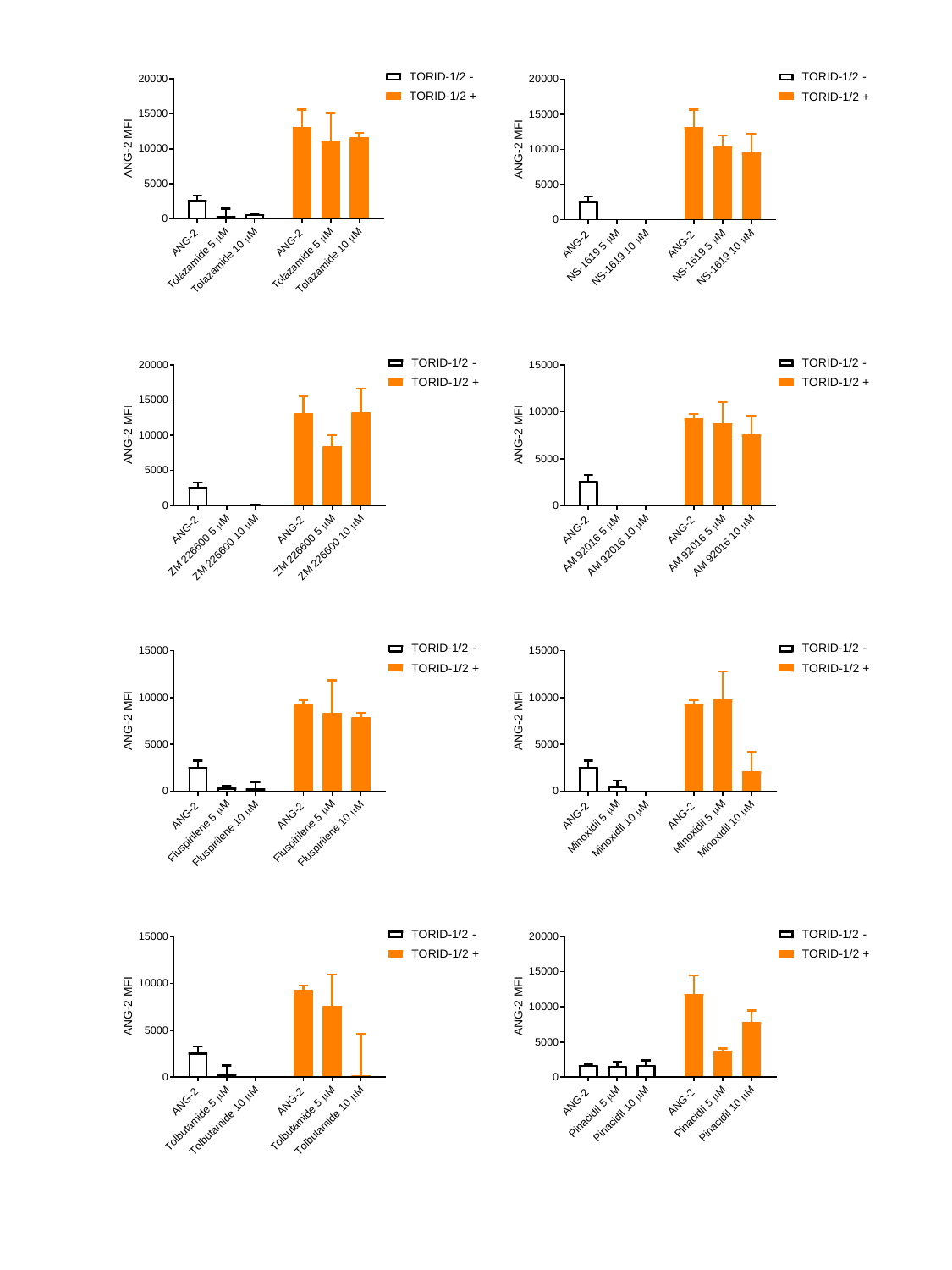

## Slide 8
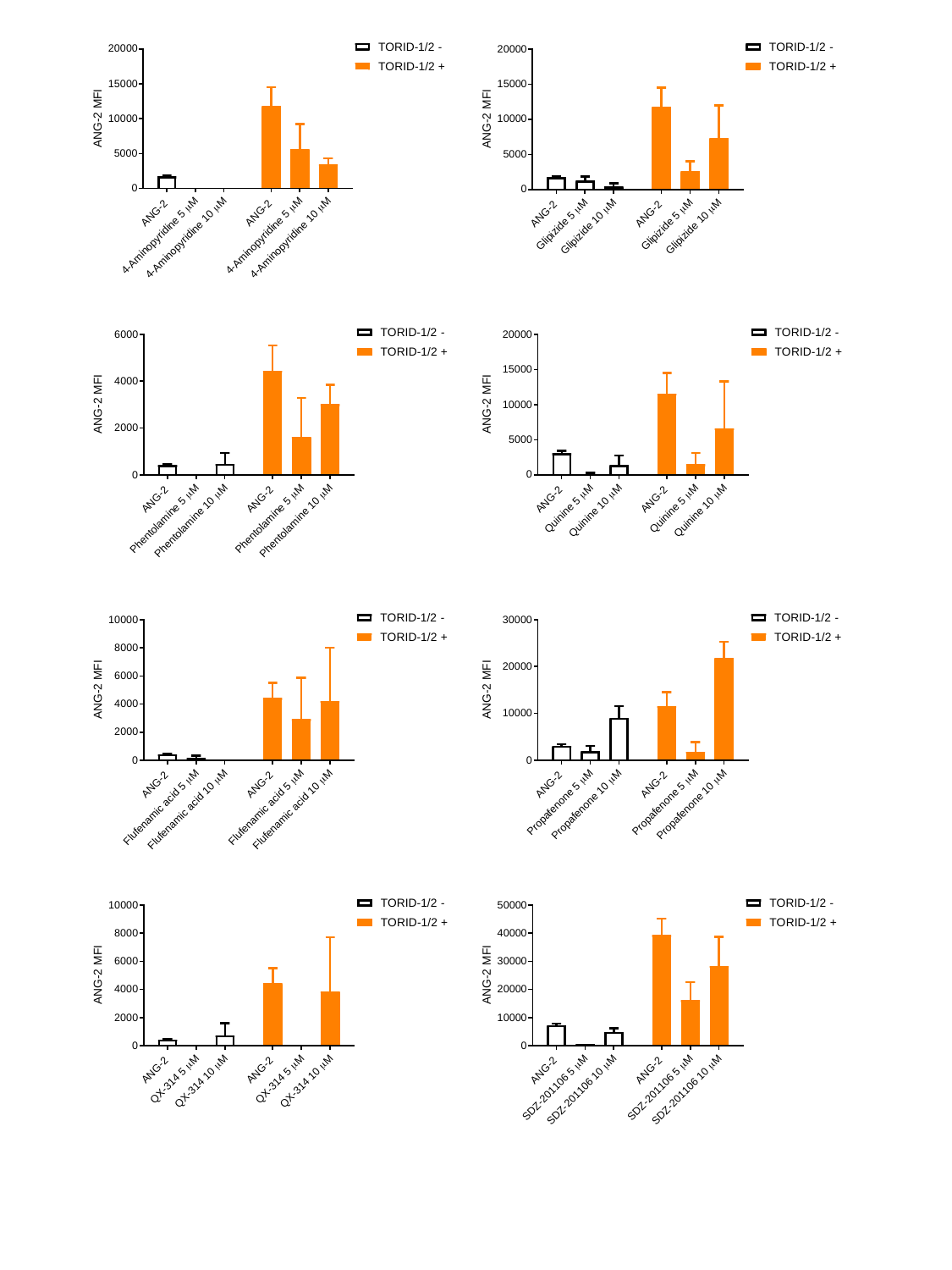

## Slide 9
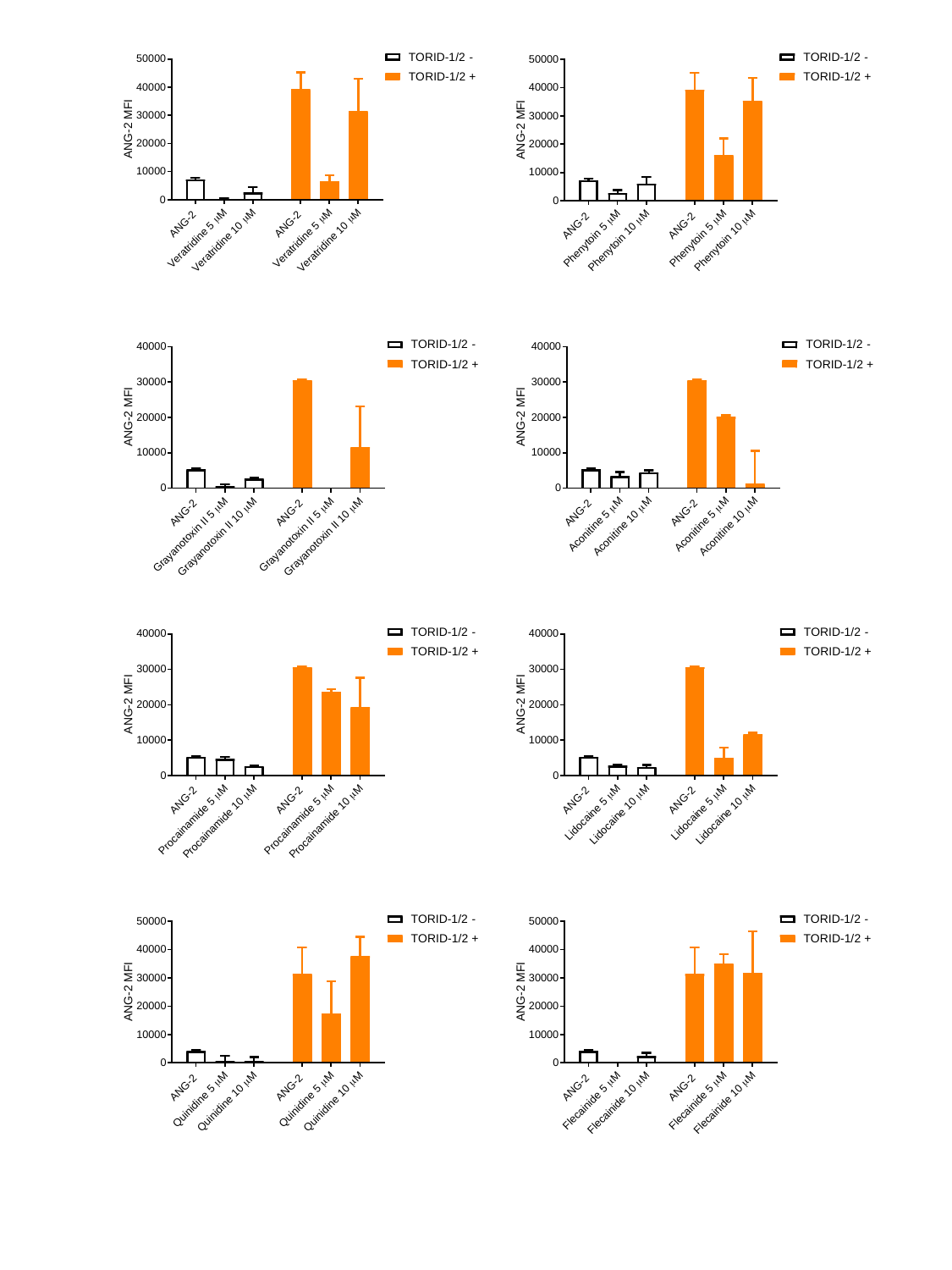

## Slide 10
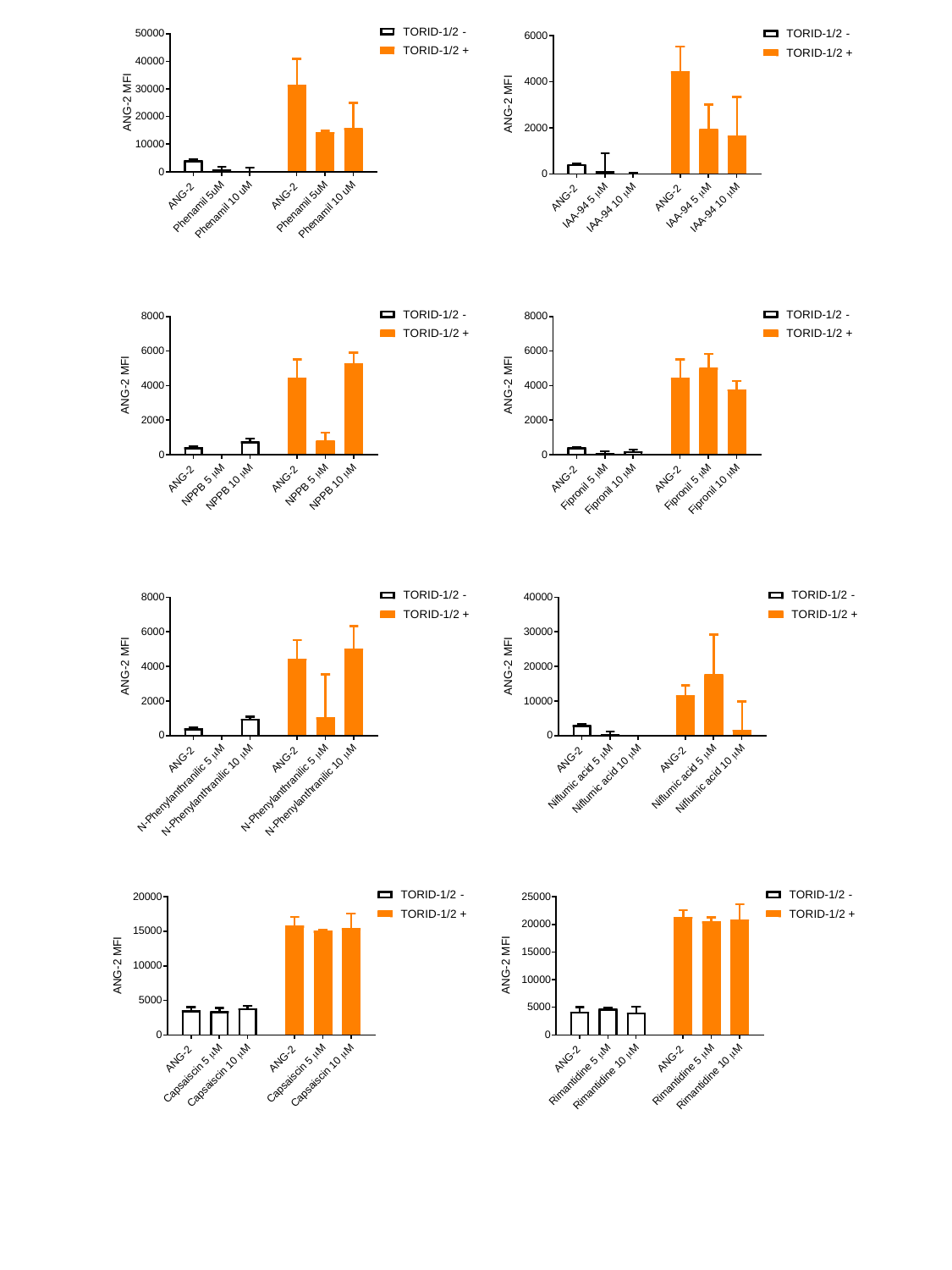

## Slide 11
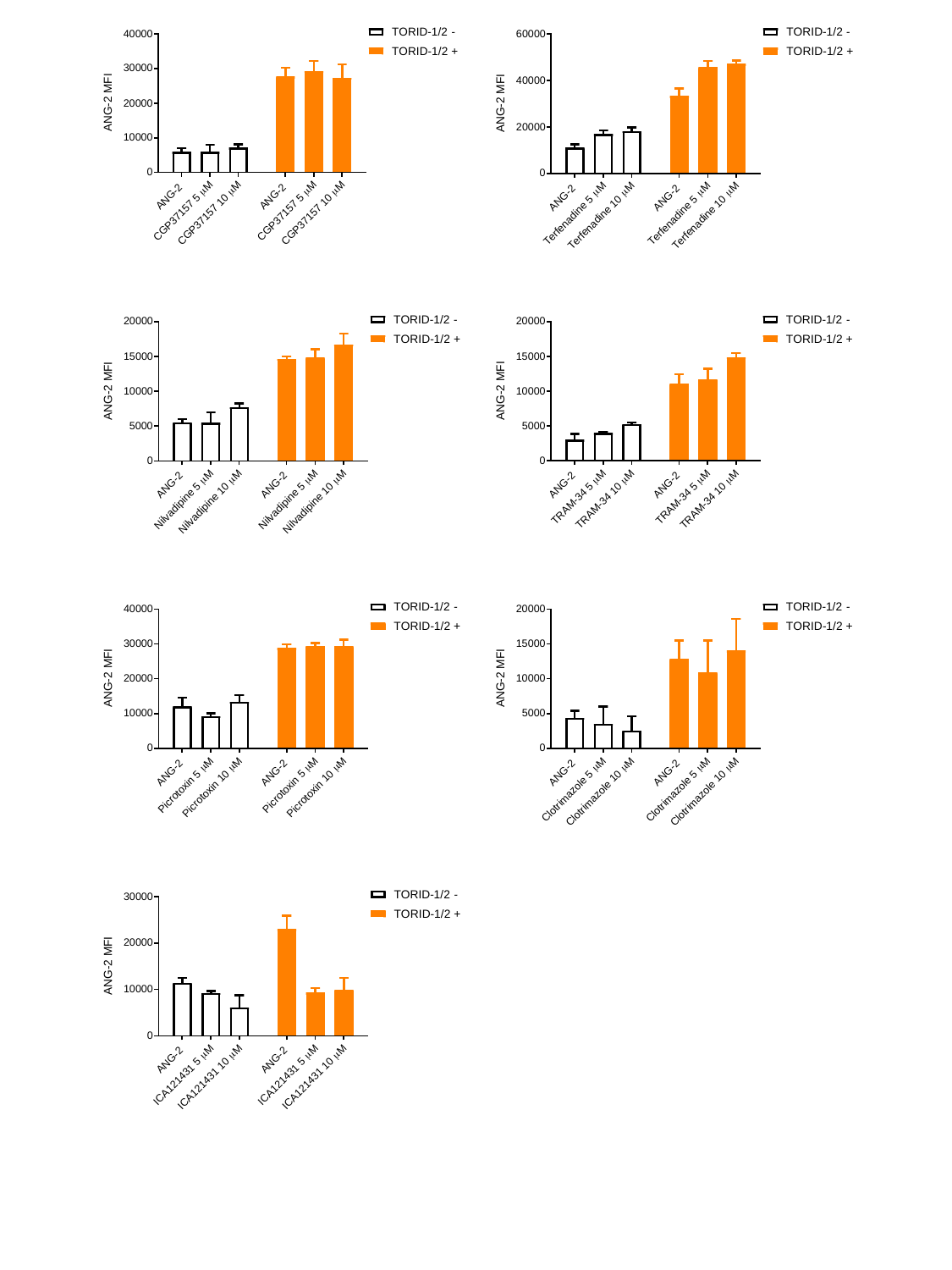

Supplement: Data S1. Screening of TMEM176B Inhibitors, Related to Figure 6 [file mmc2.zip › Submitted Supplementary Dataset 1.pptx]
